# Proton motive force partitioning links energy and redox balance to photoprotection and carbon gain

**DOI:** 10.3389/fpls.2026.1779050

**Published:** 2026-03-04

**Authors:** Fardad Didaran, Sarah MacPherson, Alice Cherestes, Saman Zohrabi, Mark Lefsrud

**Affiliations:** Biomass Production Lab, Department of Bioresource Engineering, McGill University, Lakeshore, QC, Canada

**Keywords:** ATP synthase, cyclic electron flow, cytochrome b_6_f, fluctuating light, non-photochemical quenching, pmf partitioning

## Abstract

Fluctuating irradiance forces leaves to balance energy conversion with protection against reactive oxygen species (ROS) produced when light harvesting exceeds metabolic demand. In chloroplasts, this balance is strongly governed by the thylakoid proton motive force (pmf, ΔμH^+^) and by its partitioning between a pH gradient (ΔpH) and an electric field (Δψ). A proton-circuit framework in which proton deposition by linear and cyclic electron flow builds pmf, chloroplast ATP synthase spends pmf as ATP with an effective proton conductivity g(H^+^), and counter-ion fluxes reshape ΔpH:Δψ on seconds-to-minutes timescales. Δψ-relieving anion pathways (VCCN1, CLCe) promote rapid ΔpH expression during light increases, enabling timely engagement of PsbS-dependent qE and ΔpH-dependent photosynthetic control at cytochrome b_6_f, whereas the K^+^/H^+^ antiporter KEA3 accelerates ΔpH relaxation after transitions to lower light to speed recovery. These dynamics link to stromal metabolism by describing how stromal alkalinization and Mg²^+^/thioredoxin regulation activate Calvin–Benson–Bassham enzymes, how CEF pathways (PGR5/PGRL1 and NDH) increase pmf without net NADPH production, and how phosphate recycling and triose-phosphate utilization constrain ATP synthase flux. This review examines how thylakoid architecture could generate spatial heterogeneity in proton dynamics and highlight what remains inferred versus directly measured. Finally, we present an operating-regime map and a minimal diagnostic toolkit—multiwavelength ECS (pmf, ΔpH/Δψ, g(H^+^)) combined with NPQ, P700, and gas exchange—to translate mechanism into testable predictions and improve cross-study comparability. The unifying design principle is timing: rapid ΔpH formation to protect PSI during upshifts, followed by timely relaxation to minimize unnecessary quenching and sustain CO_2_ assimilation.

## 1 Introduction

Photosynthesis converts light energy into chemical free energy, supporting most life on Earth by producing reduced carbon and O_2_ (Hohmann-Marriott and Blankenship, 2011). In leaves, the immediate energetic intermediate that links light-driven electron transfer to ATP synthesis is the proton motive force (pmf, ΔμH^+^) across the thylakoid membrane. Water oxidation at photosystem II (PSII) and proton-coupled plastoquinol oxidation at the cytochrome b_6_f complex deposit protons into the thylakoid lumen, generating a pmf with two thermodynamic components: a chemical gradient (ΔpH) and a transmembrane electric potential (Δψ). Although ΔpH and Δψ are thermodynamically interchangeable contributors to ΔμH^+^, they are not physiologically equivalent: ΔpH sets lumen pH (a chemical signal sensed by multiple regulatory modules), whereas Δψ sets the electric field across the membrane (Jagendorf and Uribe, 1966; Cruz et al., 2001). In intact leaves, pmf is often expressed predominantly as ΔpH under steady illumination, yet Δψ can contribute strongly during rapid transients or persist in contexts where counter-ion movements, buffering capacity, or membrane ion permeability constrain charge compensation (Kramer et al., 2004; Tikhonov, 2013). Thus, pmf partitioning reflects not only regulatory “intent,” but also physical and biochemical constraints that shape how quickly a given pmf can be converted into lumen acidification. pmf powers ATP synthesis by chloroplast ATP synthase and, through its partitioning between ΔpH and Δψ, influences the activation thresholds and kinetics of rapid photoprotective feedbacks *in vivo* (Avenson et al., 2005; Davis et al., 2017).

Natural irradiance is highly dynamic, especially within canopies where sunflecks and shadeflecks occur on seconds-to-minutes timescales (Kaiser et al., 2018). During rapid increases in light, electron input from the photosynthetic apparatus can temporarily exceed the capacity of stromal sinks to consume ATP and reducing power, increasing the risk of photosystem I (PSI) acceptor-side over-reduction and reactive oxygen species (ROS) formation (Havaux, 2020; Kono and Terashima, 2016). A key protective response is ΔpH-dependent “photosynthetic control” at cytochrome b_6_f, in which lumen acidification slows plastoquinol oxidation at the Qo site and restricts electron delivery to PSI when sinks lag (Tikkanen et al., 2014, 2015; Degen and Johnson, 2024). In parallel, lumen acidification activates non-photochemical quenching (NPQ)—especially the rapid, reversible qE component mediated by PsbS and supported by xanthophyll cycling—thereby reducing excitation pressure on PSII and reshaping upstream electron flow (Li et al., 2000, 2002; Fan et al., 2015). At the same time, rapid photoprotection under fluctuating light is not proton-dependent alone: antenna re-organization, protein phosphorylation/state transitions, and redox signaling can modulate excitation distribution and electron-transfer capacity in ways that interact with (but are not reducible to) ΔpH dynamics (Johnson and Ruban, 2014; Rochaix, 2014). Because PSI photoinhibition recovers slowly and can depress CO_2_ assimilation long after a transient, the timing of lumen acidification and its relaxation—together with how other fast regulatory layers are coordinated with these proton signals—is a central determinant of integrated carbon gain under fluctuating light (Zivcak et al., 2015; Kaiser et al., 2018).

Mechanistically, pmf dynamics during light transitions can be organized by three coupled levers: (i) proton deposition by linear electron flow and by cyclic electron flow (CEF) around PSI, (ii) proton efflux through chloroplast ATP synthase, and (iii) counter-ion fluxes that redistribute a given pmf between Δψ and ΔpH (Avenson et al., 2005; Davis et al., 2017). ATP synthase is not a passive consumer of pmf: redox regulation and kinetic control tune its effective proton conductivity—often parameterized as g(H^+^)—thereby setting the time constant for ΔpH decay after changes in light or metabolic demand (Kiirats et al., 2009; Hisabori et al., 2013). Conversely, stromal metabolism feeds back on proton-circuit dynamics because ATP synthase throughput depends on substrate availability, including ADP and inorganic phosphate (Pi); limitations in Pi recycling or triose-phosphate export can therefore constrain how effectively pmf is “spent” as ATP and can prolong ΔpH-dependent regulation even when light decreases (Kiirats et al., 2009). CEF adds a tunable proton flux on top of linear flow without net NADPH production; genetic and physiological studies implicate both the PGR5/PGRL1 pathway and the NDH complex in sustaining pmf and PSI safety under fluctuating light or high ATP demand (Hertle et al., 2013; Munekage et al., 2004; Hertle et al., 2013; Shikanai, 2007; Nawrocki et al., 2019; Yamori et al., 2016). Importantly, the relative dominance and physiological “purpose” of these CEF routes can differ across photosynthetic systems and cell types. For example, in C_4_ species—particularly within bundle-sheath chloroplasts—NDH-dependent CEF can be quantitatively dominant and is often interpreted primarily as an ATP-supply mechanism for the CO_2_-concentrating cycle, rather than mainly as a rapid photoprotective buffer emphasized in many C_3_ leaf contexts (Yamori et al., 2016; Ermakova et al., 2019). This altered energetic context reshapes how pmf is generated, partitioned, and read out by downstream effectors, and it motivates a cautious, context-aware interpretation of ΔpH “timing” as a design principle across diverse photosynthetic systems.

Thylakoid ion transport provides the fast counter-ion pathways needed to reshape pmf partitioning on seconds-to-minutes timescales. The bestrophin-like Cl^-^ channel VCCN1 and the CLC-family member CLCe contribute to Δψ dissipation, promoting rapid lumen acidification and timely engagement of qE and photosynthetic control during light increases (Duan et al., 2016; Herdean et al., 2016a; Dukic et al., 2019, 2022). By contrast, the thylakoid K^+^/H^+^ antiporter KEA3 accelerates ΔpH relaxation after transitions to lower light, helping qE relax and improving recovery of quantum yield when strong protection is no longer required (Armbruster et al., 2014, 2016; Wang and Shikanai, 2019). Evidence that KEA3 activity is modulated by stromal energy status reinforces that “ion transport” is not an isolated layer, but part of a feedback network linking metabolism, ATP synthase conductivity, and photoprotection (Armbruster et al., 2016; Correa Galvis et al., 2020). At the same time, the operational flexibility of ΔpH/Δψ partitioning is bounded by thylakoid permeability, buffering capacity, and charge-screening effects; these constraints can limit how independently ΔpH and Δψ can be tuned *in vivo*, especially under stress or unusual ionic/metabolic states (Kramer et al., 2004; Tikhonov, 2013). Finally, additional K^+^ pathways illustrate why transporter identity and localization must be treated cautiously: TPK3 was initially reported as thylakoid-localized, but later work found photosynthesis largely unaffected by loss of TPK3 function, consistent with a non-thylakoid role and underscoring unresolved aspects of the thylakoid ion transportome (Carraretto et al., 2013; Höhner et al., 2019).

In this review, we discuss a pmf- and partitioning-centered (“proton-circuit”) view of leaf photosynthesis that connects thylakoid biophysics (“pumps, valves, and shapers”) to photoprotection and carbon gain under fluctuating light, while explicitly noting where energetic context, membrane properties, and metabolic state can shift the dominant control points. Section 1 defines how pmf is generated and parsed into ΔpH and Δψ in intact leaves and summarizes how electrochromic-shift (ECS) methods quantify pmf, partitioning, and ATP synthase conductivity *in vivo*, including key methodological caveats (Cruz et al., 2001; Davis et al., 2017). Section 2 discusses how proton pumping (linear flow and CEF), proton efflux through ATP synthase (g(H^+^)), and counter-ion transport (KEA3, VCCN1, CLCe) interact—within physiological and biophysical constraints—to set lumen pH and PSI redox poise during light transients. Section 3 then focuses on ΔpH-sensitive effectors—PsbS-mediated qE, xanthophyll-cycle enzymes, and ΔpH-dependent photosynthetic control at cytochrome b_6_f—and emphasizes how their kinetics and amplitudes are modulated by genotype, pigment state, and environmental history (Li et al., 2000, 2002; Fan et al., 2015; Demmig-Adams et al., 2020; Degen and Johnson, 2024). Section 4 connects proton-circuit dynamics to stromal activation, ATP/NADPH balancing, and CO_2_ assimilation kinetics during sunflecks and shadeflecks, including feedbacks mediated by Pi availability and sink strength. Section 5 evaluates evidence for spatial heterogeneity (“microdomain” pmf) imposed by thylakoid architecture, distinguishing what is directly measured from what is inferred. Finally, Section 6 integrates these elements into an operating-regime map and a minimal diagnostic toolkit, and in section 7, highlights how mechanistic ordinary-differential-equation (ODE) models can complement experiments by formalizing the proton circuit, quantifying sensitivity to key parameters (e.g., ion flux conductances and g(H^+^)), and helping identify where the framework may oversimplify behavior. Guided by recent advances and persistent controversies, we organize the review around three questions: (1) what sets the dynamic ΔpH:Δψ partition of pmf in intact leaves, and how does it change across light regimes and during transients? (2) how do CEF, ATP synthase g(H^+^), and thylakoid ion transporters (KEA3, VCCN1, CLCe) interact to stabilize PSI during light fluctuations, and which interactions most strongly constrain (or enable) carbon gain? (3) what molecular features determine the pH-response curves and kinetics of PsbS-dependent qE and xanthophyll-cycle engagement, and how do these kinetics shape recovery after light decreases (**Table 1**)?

**Table 1:** Abbreviations and symbols used throughout this review and their meanings.

Abbreviation	Meaning	Abbreviation	Meaning	Abbreviation	Meaning	Abbreviation	Meaning
**A**	Net CO_2_ assimilation rate	**qI**	Photoinhibitory NPQ component	**FBPase**	Fructose-1,6-bisphosphatase	**PSII**	Photosystem II
**A_C(i)_**	Assimilation–intercellular CO_2_ curve	**qH**	Sustained NPQ component	**Fd**	Ferredoxin	**PsbS**	Photosystem II subunit S
**ATP**	Adenosine triphosphate	**OEC**	Oxygen-evolving complex	**FNR**	Ferredoxin–NADP^+^ reductase	**P700**	PSI primary donor
**CBB cycle**	Calvin–Benson–Bassham cycle	**PC**	Plastocyanin	**g(H^+^)**	Proton conductivity of ATP synthase	**RCA**	Rubisco activase
**CEF**	Cyclic electron flow	**PGR5**	Proton gradient regulation 5	**KTN**	K^+^ transport nucleotide-binding domain	**RuBP**	Ribulose-1,5-bisphosphate
**CF_1_F_0_**	Chloroplast ATP synthase	**PGRL1**	PGR5-like protein 1	**KEA3**	K^+^/H^+^ antiporter 3	**SBPase**	Sedoheptulose-1,7-bisphosphatase
**CLCe**	Chloride channel E	**Pi**	Inorganic phosphate	**LEF**	Linear electron flow	**STN7**	State transition kinase 7
**Pmf**	Protonmotive force	**pmf partitioning**	ΔpH/Δψ partitioning	**LHCII**	Light-harvesting complex II	**TPK3**	Two-pore K^+^ channel 3
**ΔpH**	Proton gradient (chemical component of pmf)	**PQ**	Plastoquinone	**NDH**	NAD(P)H dehydrogenase-like complex	**TPT**	Triose phosphate/phosphate translocator
**Δψ**	Electric potential (electrical component of pmf)	**PQH_2_**	Plastoquinol	**NPQ**	Non-photochemical quenching	**TPU**	Triose phosphate utilization
**ECSt**	ECS signal at steady state	**PRK**	Phosphoribulokinase	**qE**	Energy-dependent NPQ component	**VCCN1**	Voltage-dependent chloride channel 1
**ECS**	Electrochromic shift	**PSI**	Photosystem I	**qZ**	Zeaxanthin-dependent NPQ component	**VDE**	Violaxanthin de-epoxidase

## 2 The proton motive force: components and generation

The thylakoid proton motive force (pmf, ΔμH^+^) is the electrochemical driving force for proton movement across the thylakoid membrane. It can be decomposed into two thermodynamic components: a transmembrane electric potential (Δψ) and a pH gradient (ΔpH). Although Δψ and ΔpH are thermodynamically interchangeable contributors to ΔμH^+^, they are not physiologically equivalent. ΔpH directly sets lumen pH and thus gates multiple pH-sensitive regulatory processes (e.g., photosynthetic control at cytochrome b_6_f and the activation of NPQ components), whereas Δψ reflects the electric field across the membrane and can dominate transiently when charge compensation is delayed, potentially altering recombination pathways and ROS risk even when total pmf is similar (Kaňa et al., 2019; Cruz et al., 2001; Davis et al., 2017; Tikhonov, 2013; Kaňa et al., 2019). Importantly, pmf partitioning should not be treated as a purely deterministic “regulatory choice”: the operational ΔpH:Δψ ratio is constrained by the conductance and kinetics of counter-ion pathways (anion influx and/or cation efflux), membrane capacitance and surface charge screening, and the buffering capacity and effective permeability of the thylakoid lumen and stroma (Cruz et al., 2001; Kramer et al., 2004; Tikhonov, 2013). Consequently, while many C_3_ leaves under typical ionic conditions express a large fraction of steady-state pmf as ΔpH, Δψ can persist or remain relatively large in contexts where counter-ion flux is limiting (e.g., ion-transport mutants, altered ionic strength, or particular stress states), yielding a situation in which total pmf may be substantial but lumen acidification—and therefore engagement of ΔpH-dependent effectors—lags behind (Cruz et al., 2001; Herdean et al., 2016; Davis et al., 2017; Dukic et al., 2019). Finally, even when ΔpH forms, its regulatory “effectiveness” depends on the sensitivity and availability of downstream modules (e.g., antenna composition, PsbS abundance, xanthophyll pool state) and can be modulated by additional fast regulatory layers that are not strictly proton dependent, including protein phosphorylation/state transitions and redox signaling (Johnson and Ruban, 2014; Rochaix, 2014).

The electrochemical potential difference for protons across the thylakoid membrane can be written (with sign conventions stated explicitly) as:

(Equation 1):


$$\text{Δ}\mu_{{\text{H}}^{+}}=F\text{Δ}\psi -2.303\;RT\;\Delta \text{pH}$$

where $\text{Δ}\mu_{H^{+}}$ is the electrochemical potential difference for protons (the pmf, units of J $mol^{-1}$); F is the Faraday constant (C $mol^{-1}$); Δψ is the trans-thylakoid electric potential difference, defined as the electric potential in the lumen minus that in the stroma $\left(\psi_{\text{lumen}}\psi_{\text{stroma}}\right)$ and typically expressed in volts; R is the universal gas constant (J $mol^{-1}$ $k^{-1}$); T is absolute temperature (K); and ΔpH is the pH difference across the thylakoid membrane, defined as stroma minus lumen $\left(pH_{\text{stroma}}pH_{\text{lumen}}\right)$. The factor 2.303 converts between natural and base-10 logarithms. Recent re-analyses of ECS signals reinforce this conclusion and caution against over-attributing steady-state ECS plateaus to Δψ. Because Δψ and ΔpH are thermodynamically equivalent in the pmf, any biological process that changes charge screening or ion movement (e.g. K^+^, Cl^-^, H^+^/K^+^ exchange) will redistribute pmf between Δψ and ΔpH without necessarily changing the total $\text{Δ}\mu_{{\text{H}}^{+}}$. This is central to understanding both fast protection and ATP yield in fluctuating light (Juhaszova et al., 2022), so that at ~25 °C the chemical term contributes ~59 mV per pH unit. Because Δψ and ΔpH sum to the same total driving force, any process that changes charge screening or counter-ion movement (e.g., K^+^ fluxes, Cl^-^ conductances, and K^+^/H^+^ exchange) can redistribute a given pmf between Δψ and ΔpH without necessarily changing the total Δp, with direct consequences for both ATP synthesis (which depends on total pmf) and rapid photoprotection (which depends strongly on lumen pH and thus on ΔpH expression). This is why “pmf plateau” values and “Δψ fractions” inferred from electrochromic shift (ECS) measurements must be interpreted with care: the ECS signal primarily reports the pigment Stark effect driven by Δψ, but quantitative partitioning requires rigorous multi-wavelength controls and spectral deconvolution to avoid confounding by overlapping absorbance changes (e.g., carotenoid- and antenna-associated bands) and to avoid over-assigning steady-state ECS plateaus to Δψ when much of the steady-state pmf may reside in ΔpH (Joliot and Joliot, 2002; Davis et al., 2017; Uflewski et al., 2021; Wilson et al., 2021).

**Figure 1:**
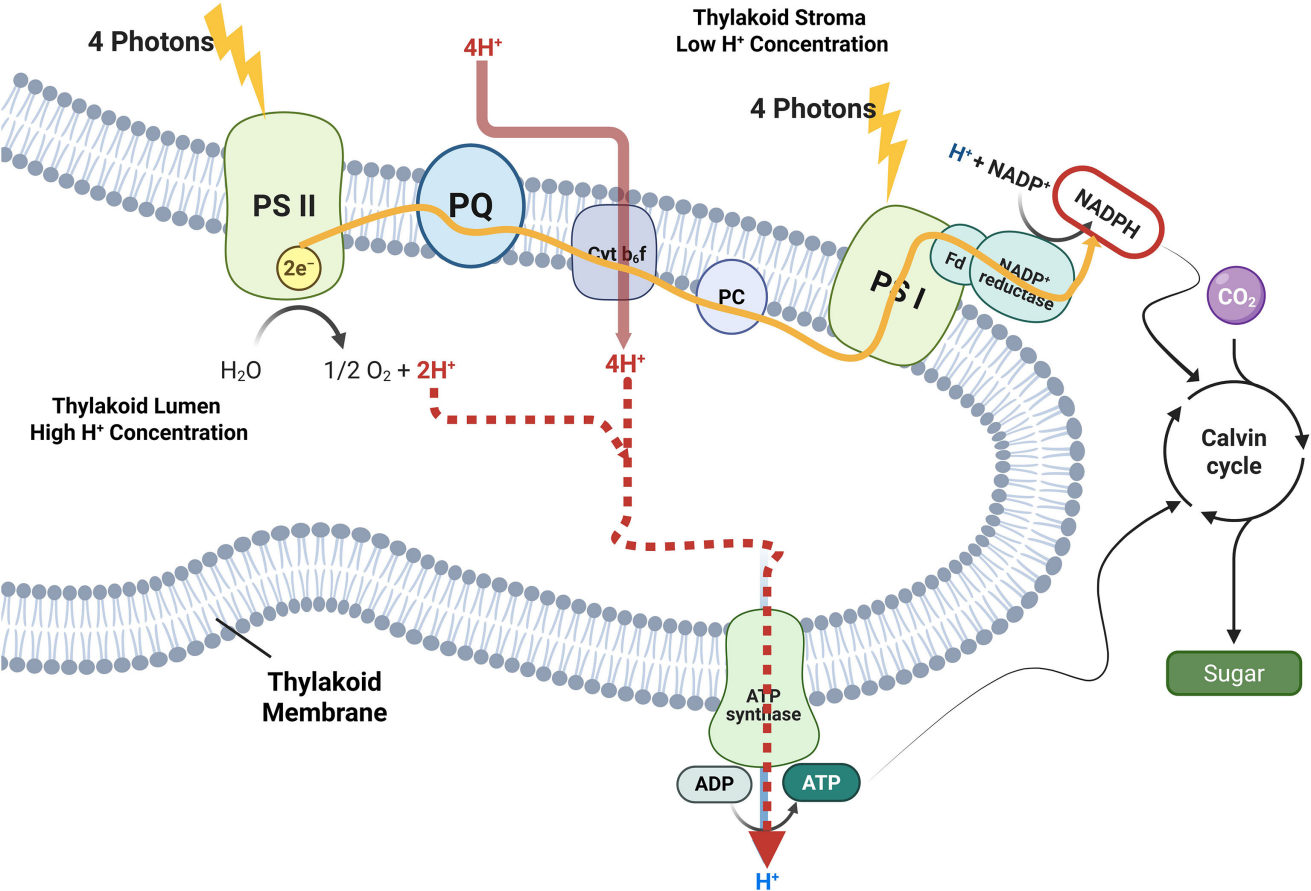
Proton-centric overview of linear electron flow and pmf formation in the thylakoid membrane. Illuminated PSII extracts four electrons from two water molecules at the oxygen-evolving complex, releasing O_2_ and four protons into the thylakoid lumen (red). Electrons reduce plastoquinone (PQ) to PQH_2_, which is oxidized at the cytochrome b_6_*f complex via the Q-cycle; this step injects additional protons into the lumen while passing electrons to plastocyanin (PC). A second photochemical lift at PSI drives ferredoxin reduction and subsequently NADP^+^ reduction by FNR to form stromal NADPH. The buildup of lumenal protons establishes the protonmotive force (pmf), composed of a chemical gradient (ΔpH) and electric potential (Δψ). Proton return to the stroma through chloroplast ATP synthase converts pmf into ATP, ATP and NADPH power CO_2_ assimilation in the Calvin cycle. Yellow paths indicate electron transfer; solid red paths indicate proton release to the thylakoid lumen; the dashed red path indicates proton return to the stroma through ATP synthase (Papageorgiou and Govindjee, 2014; Didaran et al., 2024).

The sources of proton deposition into the lumen are defined by electron flow (**Figure 1**). Water oxidation at the PSII oxygen−evolving complex releases one lumenal proton per transferred electron. Reduced plastoquinone (PQH_2_) is then oxidized at the cytochrome b_6_f complex; when the full Q−cycle operates, b_6_f contributes two additional protons per electron to the lumen (i.e., four protons per PQH_2_ oxidized), yielding a nominal total of ~3 H^+^ per electron transferred from water to PSI acceptors (Hope, 2000; Sacksteder et al., 2000; Tikhonov, 2018). Because two electrons are required to reduce one NADP^+^, this corresponds to ~6 H^+^ translocated per NADPH formed by linear electron flow (LEF). Coupled with the proton/ATP ratio of chloroplast ATP synthase (set by CF_1_CF_0_ rotor stoichiometry and potentially varying among taxa), this stoichiometric baseline helps explain why LEF alone often produces an ATP/NADPH output near ~1.3 and why auxiliary ATP−biasing routes are required when stromal demand approaches ~1.5 ATP per NADPH (Joliot and Joliot, 2002; Munekage et al., 2004; Tikhonov, 2018). Notably, these “stoichiometric constraints” provide a useful energetic scaffold, but *in vivo* outcomes can deviate from simple stoichiometry because pmf utilization depends on ATP synthase conductance and substrate availability (ADP and Pi), and because pmf partitioning (Δψ vs ΔpH) affects how quickly lumen pH reaches the thresholds for rapid regulatory feedbacks during transients (**Figure 1**).

Structural and mechanistic work has consolidated the basis for lumenal proton release and its coupling to electron transfer. High-resolution cryo-EM reconstructions of plant cytochrome b_6_f reveal quinones poised at the Qp site and features consistent with a plastoquinone channel, strengthening the mechanistic linkage between electron bifurcation in the Q-cycle and proton release/uptake across the membrane (Johnson, 2011; Tikhonov, 2018). Complementary time-resolved and mutational studies on PSII have identified hydrogen-bonded proton-egress pathways from the oxygen-evolving complex, consistent with the idea that protein architecture and lumenal microenvironment can shape how rapidly lumen acidification develops after light onset. Together, these insights rationalize why ΔpH-dependent slowdown of plastoquinol oxidation at b_6_f (“photosynthetic control”) provides a direct chemical coupling between lumen proton activity and electron-transfer throughput (Herdean et al., 2016; Davis et al., 2017; Tikhonov, 2018).

Cyclic electron flow (CEF) superimposes a tunable proton-pumping contribution on this LEF baseline without net NADPH production (Miller et al., 2022). In the PGR5/PGRL1-dependent route, electrons from the PSI acceptor side return to the plastoquinone pool, increasing b_6_f turnover and thereby increasing proton deposition to the lumen primarily through the Q-cycle (**Figure 1**). In the NDH-dependent route, a NAD(P)H dehydrogenase-like complex contributes additional proton pumping on top of b_6_f turnover, increasing the effective H^+^/e^-^ yield of cyclic flow relative to the PGR5/PGRL1 route (Shikanai, 2007; Nawrocki et al., 2019; Yamori et al., 2016). The physiological interpretation of CEF therefore depends on context: in many C_3_ leaf settings it is often discussed as supporting rapid PSI safety by strengthening pmf-linked control during upshifts, whereas in high-ATP-demand contexts—most prominently C_4_ bundle-sheath chloroplasts—NDH-dependent CEF can be quantitatively dominant and may be better viewed primarily as an ATP-supply strategy for the CO_2_-concentrating mechanism (Yamori et al., 2016; Ermakova et al., 2019). In either case, increased proton deposition via CEF does not automatically translate into faster lumen acidification: whether added pmf appears mainly as ΔpH versus Δψ depends on concurrent counter-ion conductances and on ATP synthase proton efflux at that moment. This conditionality is central for linking “pmf generation” to downstream regulation and for avoiding over-deterministic interpretations in which ΔpH timing alone is assumed to govern all protection–productivity trade-offs across photosynthetic systems.

Plant ATP synthase typically assembles a C-ring of ~14 subunits, so one full rotation returns ~14 protons and synthesizes ~3 ATP, giving ~4.67 H^+^ per ATP as a practical coupling ratio. Species variation (c_13-15_) and engineered rotor expansions illustrate the trade-off: more protons per ATP lower energy efficiency and force compensations elsewhere in the photosynthetic network, while tighter coupling raises the ATP yield per unit pmf but can limit protective ΔpH formation unless counter-ion routes keep Δψ in check (Davis et al., 2017; Roach and Krieger-Liszkay, 2019).

Pmf partitioning into ΔpH and Δψ comprise two distinct features. First, within the first minute of illumination, leaf pmf is usually carried predominantly as ΔpH, a state that is inherently safer because ΔpH activates qE and engages photosynthetic control at b_6_f, whereas sustained Δψ favors charge-recombination pathways that generate singlet oxygen at PSII. Second, interpreting Δψ from ECS requires care: the pigment Stark effect at ~515–520 nm overlaps with xanthophyll-associated bands (ΔA_505_, ΔA_535_), so quantitative partitioning demands standard protocols, spectral references, and transparent reporting to make data comparable across laboratories (Joliot and Joliot, 2002; Shikanai, 2007; Kato et al., 2012).

## 3 Dynamic partitioning of the proton motive force (ΔpH vs Δψ)

The thylakoid proton motive force (pmf, ΔμH^+^) comprises two thermodynamically interchangeable components: the transmembrane electric potential (Δψ) and the pH gradient (ΔpH). However, Δψ and ΔpH are not physiologically equivalent, because ΔpH directly sets lumen pH—the chemical signal that engages rapid photoprotective feedbacks—whereas Δψ reflects the electric field across the membrane and can dominate transiently when counter-ion fluxes lag behind proton deposition (Cruz et al., 2001; Davis et al., 2017). In intact leaves, the regulatory question is rarely “how much pmf?” alone, but how quickly pmf is built and how it is partitioned into ΔpH versus Δψ during light transitions, because that partitioning controls (i) ATP synthesis and (ii) the activation thresholds and kinetics of ΔpH-dependent effectors, including qE and photosynthetic control at cytochrome b_6_f (Davis et al., 2017; Correa Galvis et al., 2020).

A mechanistically useful way to organize pmf partitioning is by three coupled levers:

1. proton deposition (“supply”) by linear electron flow (LEF) and cyclic electron flow (CEF),
2. proton efflux (“valve”) through chloroplast ATP synthase, often summarized as an effective proton conductivity g(H^+^), and
3. counter-ion pathways (“shapers”) that dissipate Δψ and thereby allow a given pmf to be expressed more as ΔpH (Cruz et al., 2001; Davis et al., 2017).

### 3.1 Proton deposition sets the pmf supply term

On the input side, proton deposition increases with irradiance because PSII water oxidation releases protons to the lumen and cytochrome b_6_f transfers additional protons to the lumen during plastoquinol oxidation via the Q-cycle. *In vivo*, the resulting pmf rise is opposed by proton return through ATP synthase and by any dissipative leaks; thus, the instantaneous pmf reflects the net balance between proton deposition and proton efflux (Sacksteder et al., 2000; Davis et al., 2017). Importantly, the initial electrical component (Δψ) can rise rapidly at light onset or during an upshift, but counter-ion fluxes (anion influx and/or cation efflux) typically dissipate Δψ on short timescales, allowing continued proton deposition to manifest as lumen acidification (ΔpH) (Cruz et al., 2001; Davis et al., 2017).

Because many of the fastest photoprotective controls are ΔpH-dependent, not “pmf-dependent in general,” the capacity to convert Δψ into ΔpH is a key determinant of how effectively the proton circuit protects PSI during rapid light increases (Davis et al., 2017; Herdean et al., 2016b).

### 3.2 Cyclic electron flow steers pmf without net NADPH

**Figure 2:**
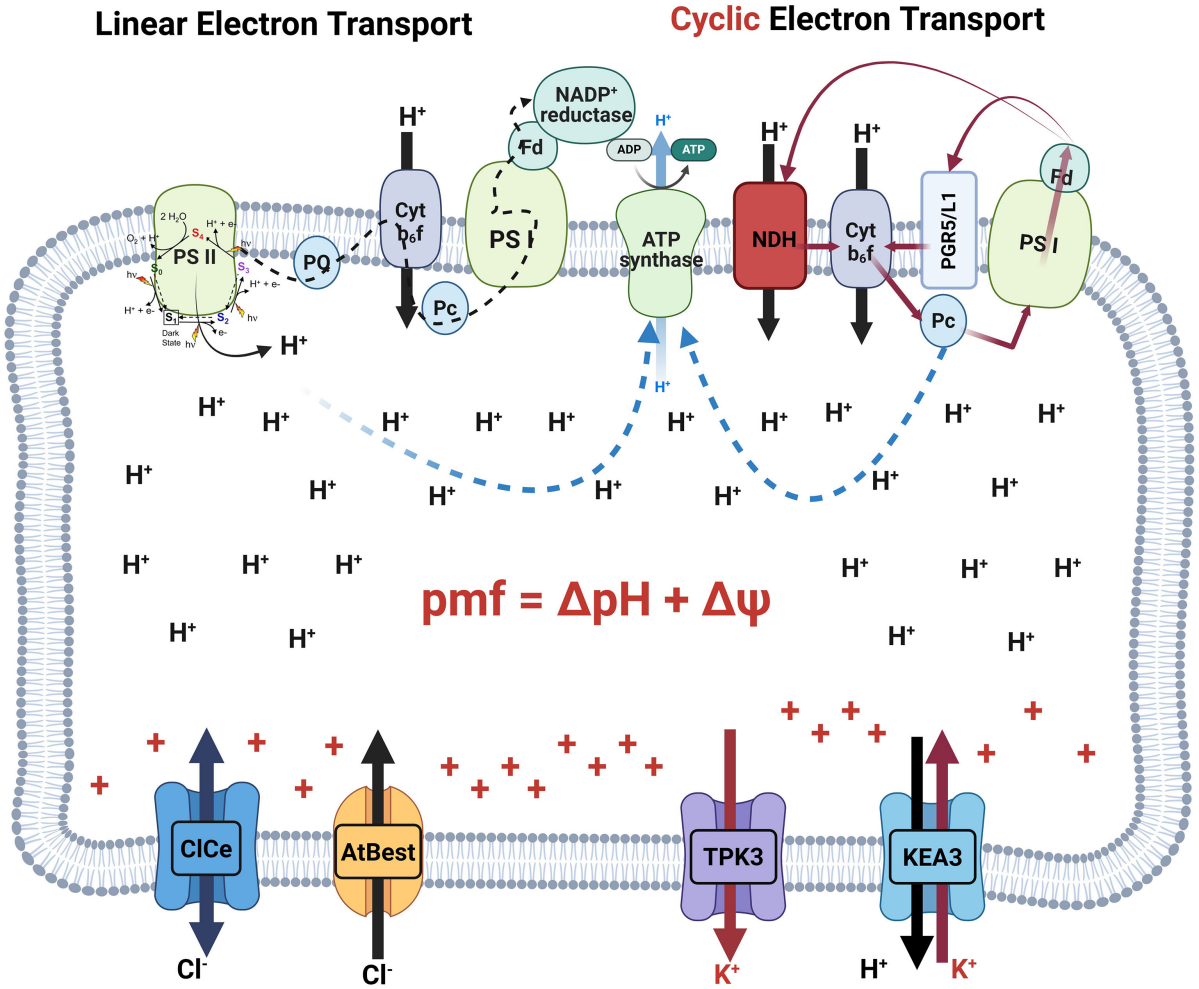
Proton-centric control of pmf partitioning in the thylakoid. Linear electron flow (left to right) oxidizes water at PSII, reduces plastoquinone (PQ), and uses the cytochrome b_6_f Q-cycle to inject protons into the lumen before electrons reach PSI and ferredoxin/NADP^+^ reductase. Cyclic electron flow around PSI occurs through the PGR5/PGRL1 route and the NDH complex (right), returning electrons to the PQ pool and b_6_f, thereby increasing proton pumping without net NADPH production (Manoj et al., 2022). The resulting proton motive force (pmf = ΔpH + Δψ) drives H^+^ return through chloroplast ATP synthase to produce ATP (blue dashed path). Partitioning of pmf is shaped by counter-ion fluxes at the membrane: VCCN1 and CLCe provide anion influx that dissipates Δψ and favors ΔpH; KEA3 exchanges stromal K^+^ for luminal H^+^ to relax ΔpH after light decreases; TPK3 is shown as a putative K^+^ channel whose thylakoid localization remains debated. By governing the balance between ΔpH and Δψ, these ion pathways determine the engagement of ΔpH-dependent photoprotective responses (e.g., PsbS-qE and ΔpH-gated control at b_6_*f) and thus the stability of PSI and ATP supply during fluctuating light (Armbruster et al., 2016; Wang and Shikanai, 2019).

CEF around PSI increases pmf without net NADPH accumulation by recycling electrons from the PSI acceptor side back to the plastoquinone (PQ) pool, increasing proton deposition predominantly through additional b_6_f turnover and (for the NDH branch) direct proton pumping (Shikanai, 2007; Nawrocki et al., 2019). Two routes are commonly distinguished: a PGR5/PGRL1-dependent pathway and an NDH-dependent pathway (**Figure 2**) (Shikanai, 2007; Nawrocki et al., 2019).

CEF returns electrons to PQ, its impact on PQ redox is conditional, reflecting the balance among electron input, b_6_f throughput, PSI acceptor-side capacity, and downstream stromal sink activity. A more defensible framing is that CEF can support PSI integrity indirectly by (i) increasing pmf/ΔpH and thereby strengthening photosynthetic control at b_6_f (slowing electron delivery to PSI when sinks lag) and/or (ii) improving ATP supply to stimulate stromal metabolism, which can accelerate electron consumption and relieve acceptor-side limitation (**Figure 2**) (Shikanai, 2007; Tikkanen et al., 2015; Davis et al., 2017; Degen and Johnson, 2024).

The key point for pmf partitioning is conditional: CEF increases the supply of pmf, but whether that increase appears mainly as ΔpH (versus Δψ) depends on counter-ion dissipation of Δψ and on ATP synthase proton efflux (g(H^+^)) at that moment (Cruz et al., 2001; Davis et al., 2017).

### 3.3 The proton efflux “valve”: ATP synthase sets how fast pmf is spent

Chloroplast ATP synthase converts pmf into ATP, but its throughput is strongly regulated. Redox regulation of the γ-subunit (a thiol/disulfide switch controlled by thioredoxin) suppresses wasteful ATP hydrolysis in the dark and modulates enzyme activity in the light, thereby changing the effective rate at which protons return to the stroma (Hisabori et al., 2013). *In vivo*, pmf discharge kinetics are often parameterized as an effective proton conductivity, g(H^+^), estimated from the electrochromic shift decay after dark intervals (DIRK/ECS approaches) (Kiirats et al., 2009; Davis et al., 2017).

For dynamic partitioning, the logic is simple but must be stated precisely:

- When g(H^+^) is high, pmf is spent rapidly, ΔpH relaxes quickly after a downshift, and ATP supply can keep pace—but ΔpH may not build as strongly during upshifts if counter-ion flux and pumping do not outpace efflux.
- When g(H^+^) is low, pmf (often ΔpH) can rise higher and persist longer, strengthening qE and photosynthetic control—but prolonged low conductance can constrain ATP delivery and delay recovery of photosynthetic yield after light decreases (Kiirats et al., 2009; Davis et al., 2017).

Crucially, g(H^+^) is not a “leaf constant”: it responds to redox regulation and also to metabolic state (ADP, Pi availability, and stromal demand), creating a feedback loop in which carbon metabolism can modulate how rapidly pmf is cashed out as ATP (Kiirats et al., 2009; Correa Galvis et al., 2020). Recent mechanistic work in algae further emphasizes that regulatory tuning of ATP synthase can differ across lifestyles and environmental regimes, reinforcing that ATP synthase control is a legitimate regulatory lever rather than a passive boundary condition (Rühle et al, 2024).

### 3.4 Counter−ion fluxes reshape partitioning by dissipating Δψ

Because Δψ and ΔpH sum to the same pmf, ion movements that dissipate Δψ can increase the fraction of pmf expressed as ΔpH, allowing faster and deeper lumen acidification for a given net proton deposition. This “Δψ-relief to ΔpH-expression” logic underlies the importance of thylakoid anion and cation pathways for rapid photoprotection during light increases (Cruz et al., 2001; Davis et al., 2017).

#### 3.4.1 Anion pathways (Δψ relief during upshifts)

The bestrophin-like channel VCCN1 provides anion influx that dissipates Δψ and thereby supports rapid ΔpH formation and timely engagement of qE and photosynthetic control during illumination (Duan et al., 2016; Herdean et al., 2016b; Dukic et al., 2019). Structural work has resolved VCCN1 as a bestrophin-like homopentamer and provides constraints for future kinetic models of its gating and conductance behavior *in vivo* (Hagino et al., 2022). In parallel, the CLC-family transporter/channel CLCe contributes to chloroplast ion homeostasis and influences photosynthetic regulation; genetic analyses link CLCe to ATP availability for LHCII phosphorylation and to acclimatory responses under particular light regimes (**Figure 2**) (Herdean et al., 2016a; Dukic et al., 2022).

#### 3.4.2 KEA3 (ΔpH relaxation after downshifts)

In contrast to VCCN1/CLCe, the thylakoid K^+^/H^+^ antiporter KEA3 predominantly functions as a ΔpH relaxer after transitions to lower light by exchanging stromal K^+^ for luminal H^+^, accelerating qE relaxation and improving recovery of photochemical efficiency when strong lumen acidification is no longer required (Armbruster et al., 2014, 2016; Wang and Shikanai, 2019). Mechanistic studies further indicate that KEA3 regulation is coupled to chloroplast energy state, reinforcing that ion transport is integrated with metabolism and ATP synthase control rather than operating independently (**Figure 2**) (Armbruster et al., 2016; Correa Galvis et al., 2020; Uflewski et al., 2021).

Single- and multi-mutant studies (vccn1, clce, kea3 combinations) support the systems-level view that plants tune Δψ dissipation and ΔpH formation/relaxation to balance rapid protection with efficient recovery under fluctuating light (Dukic et al., 2019, 2022; Gollan et al., 2023). Phenotypes under fluctuating regimes are particularly informative because they report on timing: insufficient ΔpH expression during upshifts compromises protection, whereas excessively persistent ΔpH after downshifts sustains unnecessary quenching.

TPK3 is treated as a localization cautionary case, not a settled thylakoid component. TPK3 was initially reported as thylakoid-associated with photosynthetic consequences (Carraretto et al., 2013), but later work found photosynthesis largely unaffected by loss of TPK3 function and supports a vacuolar (tonoplast) role, illustrating why transporter identity and localization must be treated conservatively in “transportome” schematics (Höhner et al., 2019).

### 3.5 Partitioning during light transients: an upshift/downshift logic

A mechanistically consistent transient picture is as follows.

#### 3.5.1 Upshifts (shade to sun; seconds)

Proton deposition rises immediately. Δψ can spike rapidly at first, but effective Δψ dissipation through anion influx (e.g., VCCN1/CLCe-dependent pathways) enables a rapid rise in ΔpH, engaging qE and ΔpH-dependent photosynthetic control at cytochrome b_6_f before PSI acceptor-side limitation becomes severe. If g(H^+^) is low enough (or transiently restricted), ΔpH rises higher and protection engages more strongly; if g(H^+^) is very high and/or Δψ dissipation is weak, ΔpH expression is delayed and PSI becomes more vulnerable (Cruz et al., 2001; Davis et al., 2017; Herdean et al., 2016b; Dukic et al., 2019).

#### 3.5.2 Downshifts (sun to shade; tens of seconds)

Proton deposition falls. Recovery requires timely relaxation of ΔpH so that qE does not persist longer than necessary. KEA3 accelerates ΔpH decay after downshifts, and ATP synthase conductance (g(H^+^)) also contributes strongly to the decay constant; together, these controls set the recovery kinetics of photochemical yield. Notably, NPQ relaxation kinetics also depend on pigment-state dynamics (e.g., zeaxanthin epoxidation) (Armbruster et al., 2016; Wang and Shikanai, 2019).

Under repeated fluctuations, loss of coordinated “shaper” function can lead to mis-timed protection (ΔpH forms too slowly at upshifts) and/or mis-timed recovery (ΔpH relaxes too slowly at downshifts), increasing PSI photoinhibition risk and depressing integrated carbon gain—phenotypes reported in combined thylakoid ion transport mutants under fluctuating light (Dukic et al., 2019; Gollan et al., 2023).

### 3.6 Contextual determinants of NPQ kinetics and amplitude

In the sections “upshift/downshift” logic to link pmf partitioning to rapid photoprotective engagement and recovery, and NPQ (especially qE) is often treated as a convenient kinetic readout of how rapidly lumen acidification is expressed and relaxed. However, NPQ is ΔpH-gated rather than ΔpH-determined: for an equivalent lumen pH trajectory, the sensitivity (pH threshold), amplitude, and relaxation of NPQ can differ substantially with genotype, pigment composition, and environmental or metabolic history. Conversely, differences in NPQ kinetics cannot be interpreted uniquely as differences in ΔpH timing unless pmf/partitioning (e.g., ECS-derived ΔpH fraction and g(H^+^)) and, where relevant, pigment state are assessed in parallel. These dependencies are especially important under fluctuating light, where “pre-conditioning” (previous sunflecks, stromal redox state, and temperature) can change the apparent speed and magnitude of NPQ independently of the underlying proton-circuit dynamics.

**Table 2** summarizes major factors that shift NPQ induction and relaxation in intact leaves, providing a practical guide for interpreting NPQ transients without implicitly assuming a uniform activation model (**Table 2**).

**Table 2:** Major factors that shift NPQ kinetics and amplitude (qE/qZ-dominated responses) beyond ΔpH timing.

Factor	Mechanistic lever (what changes)	Expected effect on NPQ induction	Expected effect on NPQ relaxation	Key references
Xanthophyll pool size/xanthophyll de-epoxidation state	Pigment availability stabilizes/strengthens quenched antenna states (qE amplitude support; qZ persistence)	Larger pool and/or pre-existing Z to higher NPQ amplitude and often faster apparent induction; additional Z accumulation during high light adds a slower (minutes) enhancement	Slower relaxation; higher carryover risk until ZE reconverts Z (minutes), especially after repeated sunflecks	Li et al. (2002); Demmig-Adams et al. (2020)
Light history/acclimation (recent sunflecks; growth light)	Changes baseline quenching competence (PsbS level, antenna organization), pre-sets xanthophyll state, and can influence phosphorylation/organization	Recent/high-light history often primes faster/higher NPQ (pre-existing Z and higher quenching competence); shade history often yields slower/lower NPQ	High-light history often causes slower relaxation and greater carryover (Z retention, sustained quenching competence); shade history tends to relax faster	Correa-Galvis et al. (2016); Demmig-Adams et al. (2020)
Stromal redox state/sink limitation	Couples metabolism to NPQ via ΔpH formation and persistence (through g(H^+^), ADP/Pi return) and redox-dependent regulation of antenna/protein networks	More reduced stroma/weak sinks often favor faster/higher NPQ by promoting stronger ΔpH and stronger engagement of ΔpH-gated switches; strong sinks often reduce NPQ at a given light	Sink limitation (and/or low Pi/slow ADP return) tends to slow ΔpH relaxation to slower NPQ relaxation and carryover; improved sinks speed recovery	Correa-Galvis et al. (2016); Kiirats et al. (2009); Armbruster et al. (2016)

### 3.7 A conceptual working model

**Figure 3:**
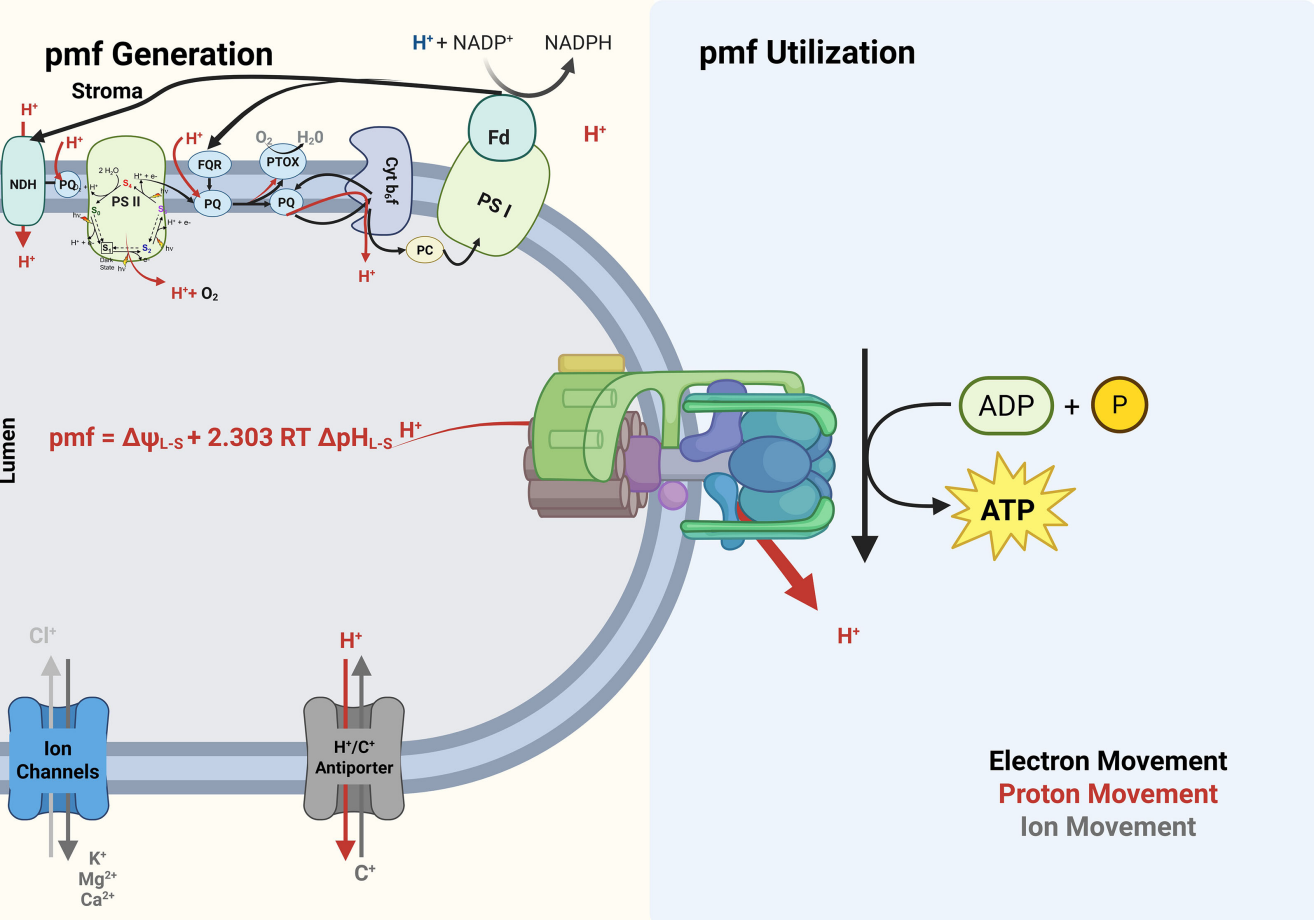
Generation and utilization of the thylakoid proton motive force (pmf). Left: pmf generation. Light energizes linear electron flow from PSII to PSI. Water oxidation at PSII releases O_2_ and protons to the lumen; reduced plastoquinone (PQH_2_) is re-oxidized at the cytochrome b_6_f complex via the Q-cycle, adding additional protons to the lumen while electrons pass to plastocyanin (PC) and then to PSI. Ferredoxin and FNR reduce NADP^+^ to NADPH (Tikhonov, 2018). The accumulation of lumenal protons establishes the pmf, which is the sum of the transmembrane electric potential (Δψ) and the chemical gradient (2.303 RT/F·ΔpH between lumen and stroma). Counter-ion transport sets pmf partitioning as anion channels (e.g., Cl^-^ routes) dissipate Δψ to favor ΔpH, whereas H^+^/cation antiporters (e.g., K^+^/H^+^ exchange) relax ΔpH (Correa Galvis et al., 2020). Right: Pmf utilization. Chloroplast ATP synthase converts pmf into chemical work by returning protons to the stroma and synthesizing ATP from ADP and Pi. The enzyme’s effective proton conductance governs how fast ΔpH decays during changes in light or metabolism, thereby influencing the balance between photoprotection and productivity (Didaran et al., 2024). Black trajectories indicate electron transfer, red trajectories indicate proton movement, and grey trajectories indicate counter-ion fluxes.

At the level of first principles, pmf (ΔH^+^) equals the sum of Δψ and the chemical term (2.303 RT/F)· ΔpH. Conceptually, Δψ is shaped primarily by VCCN1, CLCe, and cation fluxes, whereas the ΔpH term is set by the balance between proton pumps and ATPase-mediated efflux, modulated by KEA3. Operationally, pumps (LEF/CEF) set the supply of pmf, ATP synthase (g(H^+^)) sets how fast pmf is spent as ATP, and VCCN1/CLCe/KEA3 (plus other ion fluxes) shape how much of that pmf is expressed as lumen acidification and how quickly it relaxes. This partitioning tunes the engagement of ΔpH-dependent photoprotective responses (qE and photosynthetic control at b_6_f) and therefore the stability of PSI under fluctuating light (Cruz et al., 2001; Davis et al., 2017; Armbruster et al., 2016; Gollan et al., 2023) (**Figure 3**).

## 4 pH-sensitive effectors and photoprotective switches

Partitioning of pmf into ΔpH versus Δψ matters because ΔpH directly sets lumen pH, which serves as a chemical signal that engages multiple photoprotective “switches” on seconds-to-minutes timescales. These switches act on two complementary control axes: (i) excitation dissipation upstream of charge separation (NPQ, especially qE and qZ) and (ii) electron-transfer throttling within the chain (ΔpH-dependent photosynthetic control at cytochrome b_6_f). Together, they reduce excitation pressure on PSII and restrict electron delivery to PSI when stromal sinks lag, lowering the likelihood of ROS formation during rapid light changes (Papageorgiou and Govindjee, 2014; Davis et al., 2017; Degen and Johnson, 2024).

Lumen acidification should be treated as a gate rather than a single-input “driver.” For a given ΔpH trajectory, the realized amplitude and kinetics of quenching depend strongly on genotype (e.g., PsbS abundance/variants), pigment composition and xanthophyll pool state, antenna organization and membrane/lipid environment, and the recent light and metabolic history that sets stromal redox and substrate availability (Li et al., 2000, 2002; Correa-Galvis et al., 2016; Demmig-Adams et al., 2020). Moreover, fast regulatory layers are not strictly proton-dependent—such as protein phosphorylation/state transitions and redox signaling—which can reshape excitation distribution and antenna connectivity, thereby interacting with ΔpH-gated switches (Johnson and Ruban, 2014; Rochaix, 2014; Minagawa, 2011). These dependencies are particularly important when interpreting NPQ kinetics under fluctuating light, where “pre-conditioning” can shift apparent induction/relaxation even if pmf is similar.

### 4.1 PsbS couples lumen acidification to rapid, reversible qE

The dominant fast NPQ component in plants, qE, is triggered by lumen acidification and is strongly dependent on the small thylakoid protein PsbS (Li et al., 2000, 2002). PsbS is a member of the light-harvesting complex superfamily but does not act as the quencher itself, because it does not bind a stable set of antenna pigments in the way LHC proteins do. Instead, PsbS functions as a ΔpH-responsive regulatory module that alters the interaction network and conformational landscape of PSII antenna proteins, increasing the probability of forming quenched antenna states when lumen pH drops (Papageorgiou and Govindjee, 2014; Correa-Galvis et al., 2016).

Mechanistically, qE activation involves protonation of conserved lumen-exposed acidic residues in PsbS (often described as two key glutamates in Arabidopsis; residue numbering can vary depending on how the N-terminus is defined and processed). Structural and residue-level analyses support the view that protonation shifts PsbS toward conformations/interactions that favor antenna reorganization and quenching competence, rather than creating a pigment-localized quenching site within PsbS itself (Fan et al., 2015; Liguori et al., 2019). Residue-by-residue studies further suggest that additional local sequence features (e.g., lumen loop segments and hydrophobic patches on transmembrane helices) tune sensitivity and kinetics, which is important for explaining why qE can differ in amplitude and relaxation across genotypes and environments (Liguori et al., 2019; Correa-Galvis et al., 2016).

Because PsbS lacks pigments, the dominant quenching sites are thought to reside in LHCII and/or minor antenna complexes, and multiple quenching locations likely contribute depending on pigment composition and membrane state (Mozzo et al., 2008; Papageorgiou and Govindjee, 2014). Zeaxanthin is not a strict prerequisite for the fastest qE onset in all contexts, but it can substantially enhance and stabilize quenching states and alter relaxation, linking qE mechanistically to the xanthophyll cycle (Li et al., 2002; Demmig-Adams et al., 2020).

The npq4 phenotype demonstrates that loss of PsbS strongly suppresses qE and changes the dynamics of energy dissipation during light transitions (Li et al., 2000, 2002). However, PSI protection should not be attributed to PsbS/qE alone: electron-transfer control (e.g., PGR5-dependent regulation and ΔpH-dependent photosynthetic control at b_6_f) can dominate PSI safety under many fluctuating-light regimes, so claims that “PsbS loss causes PSI photoinhibition” must be framed as conditional and supported by direct data (Tikkanen et al., 2015; Kono and Terashima, 2016; Kono et al, 2022). Conversely, engineering studies show that accelerating NPQ relaxation (often involving PsbS plus additional components) can improve carbon gain under fluctuating light, whereas overly persistent quenching can reduce yield when the light regime does not demand sustained dissipation (Kromdijk et al., 2016; Kromdijk and Walter, 2023).

### 4.2 The xanthophyll cycle sets pigment−state amplitude and persistence of NPQ

The xanthophyll cycle couples lumen acidification to reversible changes in carotenoid composition that modulate both the magnitude and persistence of NPQ. Under acidic lumen pH, violaxanthin de-epoxidase (VDE) is activated on the lumenal side and uses ascorbate to convert violaxanthin to antheraxanthin and zeaxanthin, enriching the antenna with pigments that stabilize quenching states and improve photoprotection (Demmig-Adams et al., 2020). Importantly, VDE activity is not only pH-dependent but also lipid-dependent; evidence and models support recruitment/activation in MGDG-rich membrane environments, making local lipid organization a plausible contributor to heterogeneity in quenching competence (Goss and Latowski, 2020).

During lower light or recovery phases, zeaxanthin epoxidase (ZE) reconverts zeaxanthin toward violaxanthin on the stromal side, promoting relaxation. Under photoinhibitory stress, ZE abundance or activity can decrease, prolonging zeaxanthin retention and thereby extending the lifetime of quenching states—an effect consistent with ZE degradation under PSII photoinhibition conditions (Bethmann et al., 2019; Demmig-Adams et al., 2020).

Functionally, the division of labor is best stated explicitly:

- PsbS provides fast ΔpH-gated switching (seconds) that defines the rapid onset and reversibility of qE.
- Zeaxanthin modulates amplitude and persistence (minutes), contributing to qZ-like behavior and slower relaxation when zeaxanthin remains elevated (Li et al., 2002; Demmig-Adams et al., 2020).

Thus, changes in NPQ kinetics that are attributed solely to zeaxanthin should be interpreted carefully unless PsbS status and ΔpH dynamics are simultaneously measured.

### 4.3 ΔpH-gated photosynthetic control at cytochrome b_6_f

Lumen acidification also enforces a rapid feedback at the main electron-transfer bottleneck: the cytochrome b_6_f complex. When light harvesting transiently exceeds stromal capacity to consume ATP and reducing power, ΔpH can slow plastoquinol oxidation at the Qo site (“photosynthetic control”), restricting electron transfer from PSII toward PSI and thereby reducing the probability of PSI acceptor-side over-reduction during transients (Tikkanen et al., 2015; Kono and Terashima, 2016; Degen and Johnson, 2024).

This control is mechanistically distinct from qE:

- qE reduces excitation pressure (less energy into PSII antennae).
- photosynthetic control reduces electron pressure (less electron delivery into PSI when sinks lag).

Both mechanisms are triggered by ΔpH, the timing and amplitude of lumen pH changes—set upstream by proton deposition, ATP synthase g(H^+^), and counter-ion fluxes—determine how effectively these switches engage during a sunfleck and how quickly they relax during a shadefleck (Davis et al., 2017; Degen and Johnson, 2024).

### 4.4 Slower and sustained quenching modes where pH is necessary yet not sufficient

Beyond qE, additional NPQ components operate on longer timescales and should be treated as mechanistically distinct rather than as “slower qE.”

- qZ (minutes): Zeaxanthin-associated quenching that overlaps with qE but persists longer as pigment state changes and relaxes more slowly than ΔpH alone (Demmig-Adams et al., 2020; Papageorgiou and Govindjee, 2014).
- qI (hours): Photoinhibitory down-regulation linked to PSII damage/repair dynamics rather than to a reversible ΔpH switch; it reflects sustained stress and recovery processes (Roach and Krieger-Liszkay, 2014).
- qH (hours to days): A sustained, PsbS-independent quenching mode requiring the plastid lipocalin LCNP and antagonized by SOQ1; recent work identifies LHCII trimers as sites associated with qH-type dissipation (Malnoë, 2018, 2018; Bru et al., 2022).

### 4.5 Temporal design principles linking ΔpH dynamics to effector kinetics

The system performs well when ΔpH kinetics align with the response windows of the effectors:

- Seconds (sunfleck onset): ΔpH rises rapidly; PsbS protonation enables fast qE engagement, and photosynthetic control at b_6_f can restrict electron delivery to PSI if sinks lag.
- Minutes (continued high light or repeated sunflecks): Sustained ΔpH supports VDE activity and zeaxanthin accumulation, increasing quenching magnitude and persistence (qZ-like behavior).
- Hours to days (chronic stress): qI and/or qH can dominate; these states protect but must be resolved to avoid long-lasting suppression of photochemical yield after conditions improve (Roach and Krieger-Liszkay, 2014; Malnoë, 2018).

Therefore, upstream partitioning controls—ATP synthase g(H^+^), KEA3-dependent ΔpH relaxation after downshifts, and Δψ-relieving anion pathways (VCCN1/CLCe)—indirectly determine when PsbS and VDE engage and how long quenching persists. This explains why thylakoid ion transport mutants can show large changes in NPQ amplitude and relaxation even when antenna proteins are unchanged (Davis et al., 2017; Armbruster et al., 2016; Dukic et al., 2019, 2022).

## 5 From protons to carbon: how ΔpH and Δψ shape CO_2_ assimilation and metabolism

Everything upstream in the proton circuit—pmf production, its ΔpH:Δψ partitioning, and pH-sensitive switches—exists to stabilize carbon fixation and the broader metabolic network under real, fluctuating environments (Yamori et al., 2016; Gollan et al., 2023; Wu et al., 2024; Moland et al., 2025). In this section, lumen acidification and stromal alkalization are connected to activation of the Calvin–Benson–Bassham (CBB) cycle, to ATP/NADPH balancing via cyclic or alternative electron routes and metabolite shuttles, in addition to the dynamic control of assimilation during sun- and shade flecks (Davis et al., 2017).

### 5.1 Light-driven pH changes “switch on” the CBB cycle-but flux is gated by adenylates and Pi

Light-driven proton pumping acidifies the lumen while alkalinizing the stroma, and this pH reset is not ancillary; it is required to bring several CBB enzymes into their high-activity regime and to coordinate them with the light reactions (Walker et al., 1986; Wang and Portis, 2006; Gregory et al., 2024). *In vivo* analyses and synthetic views converge on the point that stromal alkalization, with concomitant Mg²^+^ release into the stroma, promotes activation of FBPase, SBPase, and PRK, facilitates reductive activation by the thioredoxin system so that carbon-fixation capacity tracks light availability (Okegawa and Motohashi, 2015). Rubisco activase (RCA) links this protonic state to rubisco engagement, because its ATP/ADP- and redox-sensitive regulation makes it responsive to the chloroplast energy/redox state established by pmf and electron transport. Mechanistic and structural evidence further supports a model in which redox control tunes RCA nucleotide sensitivity, providing a molecular basis for light-dependent Rubisco activation (Wang and Portis, 2006). The proton circuit, therefore, does not merely supply ATP; it reconfigures the stromal microenvironment—pH, Mg²^+^, redox—so that the CBB cycle runs at the “right” set point for the current light and can be quickly dialed back when light fades (Werdan et al., 1975).

The “switch-on” logic is incomplete if treated only as pH/Mg²^+^/redox. ATP synthase requires Pi as a substrate, and its throughput is constrained by the return of ADP and Pi from stromal metabolism. When Pi availability is low (e.g., because Pi is sequestered in phosphorylated intermediates, export is restricted, or TPU limitation develops), proton efflux through ATP synthase can be throttled even if pmf is high, strengthening ΔpH-dependent regulation and slowing relaxation after downshifts (Kiirats et al., 2009; McClain et al., 2023). In this view, stromal alkalinization and enzyme activation set the 3.62.6 Contextual determinants of NPQ kinetics and amplitude for carbon fixation, whereas adenylate/Pi supply and sink turnover set the realized flux and strongly influence the kinetics of recovery after fluctuations.

### 5.2 Matching supply and demand: ATP/NADPH economics

By stoichiometry, LEF (Linear Electron Flow) provides ~1.3 ATP per NADPH, whereas C_3_ carbon fixation plus photorespiration often demands more ATP per NADPH, especially in warm, variable light (Cruz et al., 2001). This mismatch necessitates pmf-boosting and ATP-biasing mechanisms—most notably CEF around PSI and NDH-dependent routes—which add protons, and thus ATP potential, without net NADPH production (Cruz et al., 2001; Shikanai, 2007; Buchanan, 2017). A recent review on “alternative power lines” argues that multiple auxiliary routes—CEF (Cyclic electron flow) via PGR5/PGRL1, NDH, and photorespiratory or respiratory couplings—dynamically rebalance ATP/NADPH to sustain carbon metabolism when LEF alone would undersupply ATP (Davis et al., 2017; Kedem et al., 2021). In C_4_ leaves, where bundle-sheath ATP demand is higher due to the CO_2_-concentrating cycle, NDH-mediated CEF has been quantified *in vivo* as the dominant PSI electron route in bundle-sheath chloroplasts, underscoring the centrality of proton-pumping routes for sustaining assimilation in these tissues (Ermakova et al., 2019). When reductant accumulates (high NADPH/low ATP), chloroplasts export reducing equivalents via the NADP-MDH “malate valve,” handing off redox to the cytosol or mitochondria and indirectly helping recycle NADP^+^ for PSI while supporting ATP production outside the chloroplast; this shuttle is repeatedly highlighted as a key buffer that couples the proton circuit to downstream metabolism (**Figure 3**) (Scheibe, 2004; Selinski et al., 2014; Selinski and Scheibe, 2019).

### 5.3 Sunflecks, shadeflecks, and the shape of assimilation transients: coordinating protection with metabolic induction and Pi recycling

In natural light, assimilation rarely reaches steady state, and the timing of ΔpH formation and relaxation—set by pumps, the ATP synthase “valve,” and counter-ion routes—gates both protection and productivity on sub-minute scales (Werdan et al., 1975; Michelet et al., 2013; Savage et al., 2016; Walker et al., 2020). At sunfleck onset (low to high light), a rapid ΔpH spike triggers qE and photosynthetic control at b_6_f, preventing PSI over-reduction while Rubisco and RCA catch up; because PSI lacks a rapid repair cycle, this prioritization averts damage that would depress CO_2_ uptake for days. At shadefleck onset (high to low light), the rate of ΔpH relaxation limits recovery of PSII quantum yield and thus CO_2_ assimilation; KEA3, a thylakoid K^+^/H^+^ antiporter whose C-terminus senses chloroplast energy state, accelerates qE and ΔpH relaxation and improves post-shadefleck efficiency. Under chronic fluctuation, insufficient biasing of ΔpH at upshifts—due to weak anion or cation counter-fluxes—leads to PSI photoinhibition, slow recovery, and assimilation constraints that far outlast the transient; recent syntheses and experiments place b_6_f “photosynthetic control” at the center of this protective logic (Werdan et al., 1975; Avenson et al., 2005; Hisabori et al., 2013; Davis et al., 2017; Foyer and Noctor, 2020). Faster assimilation transients are observed when sink strength rises; for example, elevated CO_2_ lowers PSI over-reduction during light transitions by pulling electrons into the CBB cycle more rapidly (Kono and Terashima, 2016).A critical addition is that Pi recycling can be rate-limiting in both directions. During rapid induction, Pi availability influences ATP synthase throughput and therefore the pace at which ATP supply rises to support CBB activation and RuBP regeneration (Kiirats et al., 2009). During recovery, Pi sequestration in phosphorylated intermediates and limitations in export/end-product use can slow ATP synthase flux and prolong ΔpH, delaying NPQ relaxation and suppressing assimilation even when incident light has decreased (McClain and Sharkey, 2019; McClain et al., 2023). These effects help explain why assimilation transients often reflect combined “biophysical timing” (ΔpH dynamics) and “biochemical timing” (Pi return and sink engagement), and why interpreting NPQ kinetics as a direct proxy for ΔpH timing can be misleading without concurrent assessment of pmf partitioning and Pi/export status.

### 5.4 Partitioning controllers as metabolic levers

ΔpH is the shared trigger for qE, xanthophyll cycling, and b_6_f control, the same levers that set pmf partitioning, also tune CO_2_-uptake dynamics (Michelberger et al., 2025). Lower ATP synthase conductance (g(H^+^)) preserves ΔpH for protection but can transiently restrict Φ_PSII_ and ATP supply, whereas higher conductance speeds ATP delivery and ΔpH collapse but risks PSI if electron sinks lag; the optimal g (H^+^) depends on the light-fluctuation pattern and downstream sink capacity (Michelet et al., 2013; Johnson et al., 2014; Davis et al., 2017). KEA3 modulates ΔpH relaxation in response to stromal energy state (ATP/ADP, pH) and thereby helps avoid “over-holding” qE after shadeflecks, reducing unnecessary losses in ΦPSII and boosting assimilation (Armbruster et al., 2016; Wang and Shikanai, 2019). Rapid Δψ relief through VCCN1 biases pmf to ΔpH at sunflecks, strengthening protective throttling at b_6_f and preserving PSI, which maintains assimilation capacity over hours to days. The overarching design principle is that the proton circuit must be tuned not for maximal ΔpH or maximal ATP alone, but for the right ΔpH at the right time, preserving PSI while minimizing unnecessary qE carry-over that would tax ΦPSII and CO_2_ uptake (Wilson et al., 2021; Hagino et al., 2022).

### 5.5 Envelope transport, phosphate balance, and TPU

Downstream of the CBB cycle, the triose-phosphate/phosphate translocator (TPT) exchanges triose-P for inorganic phosphate (Pi), thereby coupling chloroplast export to Pi recycling for ATP synthase. Under high sugar build-up or low Pi return, leaves can enter triose phosphate utilization (TPU) limitation, in which assimilation is capped by end-product processing or export rather than by Rubisco activity or RuBP regeneration. Recent physiological and modeling studies treat TPU as a systems-level property of the source–sink network that becomes especially prominent under specific combinations of CO_2_, temperature, and sink strength, rather than as a simple, static biochemical constant (Quick and Schaffer, 2017; McClain and Sharkey, 2019; Rogers et al., 2021). Because ATP synthase requires Pi as substrate, Pi availability—and thus TPT-mediated return—feeds back onto ATP production from pmf, providing another route by which carbon-export status influences how proton-motive energy is cashed out (Cruz et al., 2001; Golding and Johnson, 2003).

Beyond transport stoichiometry, source–sink status is coordinated by broader regulatory networks that couple carbohydrate availability, phloem transport capacity, and stress responses. While a full treatment is beyond the scope of this review, the central implication for a proton-circuit framework is that carbon-status signaling and sink strength can modulate the same proton-circuit variables that determine ΔpH timing, including g(H^+^), the persistence of ΔpH, and the degree to which photoprotective responses are maintained across repeated fluctuations (Savage et al., 2016; Quick and Schaffer, 2017). Likewise, photorespiration reshapes ATP demand and carbon flux partitioning, altering when ATP supply versus protection becomes the dominant constraint. These couplings motivate interpreting “proton-centric” diagnostics (ECS-based pmf/partitioning and g(H^+^), NPQ, P700) alongside carbon-status indicators (gas exchange, ACi/TPU signatures, and—where feasible—pigment state and Pi/nutrient context) to attribute limitations correctly in intact leaves (Walker et al., 2020; Rogers et al., 2021; McClain et al., 2023).

## 6 Spatial–temporal organization and heterogeneity of proton dynamics

### 6.1 Thylakoid architecture creates functional zones and source–sink separation

Plant thylakoids form a connected membrane network organized into appressed grana stacks and unappressed stroma lamellae, with grana margins providing curved transition regions between the two. This architecture is associated with lateral segregation of photosynthetic complexes: PSII–LHCII is enriched in grana cores, whereas PSI and chloroplast ATP synthase are largely excluded from grana cores and enriched in stroma-exposed membranes; cytochrome b _6_ f is distributed across domains and has frequently been reported to show enrichment toward grana margins (Daum and Kühlbrandt, 2011; Anderson et al., 2012; Kirchhoff, 2013, 2014).

A key point for proton dynamics is that the lumen is topologically continuous, so lateral equilibration of lumen protons is possible in principle. However, continuity does not imply instantaneous equilibration: electron-tomography and diffusion-based frameworks emphasize that narrow junctions at margins, tortuous geometry, and crowding can slow effective diffusion and create conditions where local proton chemistry differs transiently across domains (Daum and Kühlbrandt, 2011; Kirchhoff, 2014; Kirchhoff et al., 2008).

### 6.2 Why local pmf can differ: lateral heterogeneity of ΔpH and Δψ

The simplest physical reason to expect heterogeneity is spatial separation of proton sources and sinks. PSII and cytochrome $b_{6}$ f deposit protons into the lumen, while ATP synthase provides the dominant proton return (“valve”), and these components are not co-localized (Kirchhoff, 2013, 2014). During light transients, this source–sink separation can generate lateral gradients in proton concentration and local pmf partitioning (ΔpH vs Δψ), especially when counter-ion pathways that dissipate Δψ are locally limiting. In this view, domain-level heterogeneity is expected to be most pronounced on seconds-to-minutes timescales where pumping/efflux rates change quickly (Tikhonov, 2012; Davis et al., 2017).

A second contributor is the local balance of charge compensation. Experiments and theory show that ionic strength and counter-ion movements can change how pmf is expressed as Δψ versus ΔpH, even when total pmf is similar (Cruz et al., 2001; Davis et al., 2017). While most measurements of Δψ/ΔpH are spatially averaged, the same logic implies that regional differences in ion permeability (anion influx and cation efflux pathways) could yield domain-dependent partitioning during transients (Davis et al., 2017).

### 6.3 Light-driven remodeling: state transitions and osmotic mechanics

Thylakoid organization is not static. State transitions remodel antenna connectivity and excitation distribution on minute timescales through STN7-dependent LHCII phosphorylation, changing the coupling between PSII and PSI and altering the spatial pattern of excitation pressure (Minagawa, 2011; Grieco et al., 2012; Mekala et al., 2015). Recent work also indicates additional STN7-linked regulatory behaviors beyond the simplest “LHCII moves” cartoon, reinforcing that multiple remodeling routes can coexist (Węgrzyn et al., 2022). Because excitation distribution helps set where electron flow (and thus proton deposition) is strongest, these reorganizations are expected to influence proton budgets indirectly, even if pmf itself remains globally dominated by ΔpH. Modeling frameworks for state transitions and membrane interactions provide useful constraints on how these reorganizations can change effective connectivity and diffusion geometry (Wood and Johnson, 2020).

In parallel, lumen swelling and osmotic responses accompany illumination and lumen acidification. Water flux following ion movements changes lumen dimensions and can therefore change effective diffusion distances and buffering capacity, implying that proton equilibration kinetics are themselves dynamically modulated by the light state (Tikhonov, 2012; Kaňa et al., 2016).

### 6.4 Where the ion-flux “shapers” sit: localization is the missing constraint for spatial models

ATP synthase is concentrated in stroma-exposed membranes and is largely excluded from grana cores, establishing spatially localized proton efflux zones that are separated from the strongest PSII proton sources in grana. This geometry alone makes transient lateral ΔpH gradients plausible unless fast charge-compensating ion fluxes minimize local Δψ buildup (Kirchhoff, 2013, 2014; Davis et al., 2017).

For ion pathways that reshape partitioning, molecular identity is now strong, but sub-domain localization is still a limiting uncertainty for spatial models. VCCN1 has been structurally resolved as a bestrophin-like homopentamer, but the *in vivo* distribution across grana margins versus stroma lamellae is not yet firmly mapped; because VCCN1 contributes to Δψ relief and supports rapid ΔpH expression during illumination, its placement will strongly influence whether ΔpH rises quickly at the relevant sites during upshifts (Herdean et al., 2016b; Dukic et al., 2019; Hagino et al., 2022). CLCe is genetically linked to chloroplast ion homeostasis and to regulatory responses that include LHCII phosphorylation behavior, but its spatial deployment across domains also remains uncertain, again limiting predictive modeling of Δψ dissipation in space (Herdean et al., 2016a; Dukic et al., 2022). KEA3 is best treated as a ΔpH relaxation module after downshifts; mapping where KEA3 is enriched (margins vs lamellae vs broader distribution) is essential for predicting where ΔpH relaxation initiates and how quickly it propagates (Armbruster et al., 2016; Wang and Shikanai, 2019).

A conservative, testable working hypothesis is that grana margins act as regulatory “exchange zones” because they juxtapose (i) a transition region between grana and lamella membranes, (ii) cytochrome $b_{6}$ f distribution that often shows margin bias, and (iii) proximity to ATP synthase-rich stroma-exposed regions. If Δψ-relieving pathways (e.g., VCCN1/CLCe) are also enriched at margins, this would favor rapid ΔpH expression during upshifts and rapid engagement of ΔpH-dependent controls. This remains a hypothesis until localization and local-pH readouts are directly measured (Kirchhoff, 2013, 2014; Davis et al., 2017; Hagino et al., 2022).

Finally, the TPK3 localization debate provides a cautionary precedent: putative thylakoid channels should not be treated as established spatial players without convergent localization and functional evidence (Carraretto et al., 2013; Höhner et al., 2019).

## 7 A unified proton-centric framework

### 7.1 The minimal dynamical model (five nodes, two loops)

At seconds-to-minutes timescales, leaf photosynthesis is well described by a five-node circuit organized into two nested feedback loops (Kiirats et al., 2009; Hohmann-Marriott and Blankenship, 2011). Proton-pumping modules, PSII, cytochrome b_6_f, and CEF, raise the total pmf. Counter-ion pathways—notably KEA3 on the K^+^/H^+^ exchange side and VCCN1/CLCe on the anion side—reshape that pmf toward its chemical component (ΔpH) by relieving the electrical component (Δψ) (Dukic et al., 2022; Hagino et al., 2022). ATP synthase then spends pmf as ATP, and its effective proton conductance, ${\text{g}}_{{\text{H}}^{+}}$, sets the rate at which ΔpH collapses after a change in light or metabolic demand. pH-sensitive effectors read the ΔpH signal and throttle photochemistry in real time: PsbS mediates rapid, reversible qE; the VDE/ZE (violaxanthin de-epoxidase/zeaxanthin epoxidase) xanthophyll cycle establishes the longer-lived, zeaxanthin-dependent quenching state; and ΔpH-gated “photosynthetic control” at b_6_f slows plastoquinol oxidation when sinks lag. Carbon metabolism (the Calvin–Benson–Bassham enzymes together with Rubisco activase and export/transport steps) draws down electrons and ATP and thereby feeds back on the upstream redox and energy state that determines how much pmf is built and how it is partitioned. In compact terms, the fast protective loop raises ΔpH, engages PsbS and *b*_6_f control, and keeps PSI oxidized, whereas the productivity loop converts pmf into ATP, activates the CBB cycle, strengthens sinks, and relieves upstream over-reduction (Werdan et al., 1975; Zu Tittingdorf et al., 2004; Dall’Osto et al., 2012; Carmo-Silva and Salvucci, 2013; Hisabori et al., 2013; Wilson et al., 2021; Carbonera et al., 2022; Juhaszova et al., 2022).

The operational objective of the chloroplast proton-control network is conserved across genotypes and environments: to generate the right ΔpH at the right time. Too little ΔpH during a light upshift delays engagement of qE and cytochrome b_6_f–dependent photosynthetic control, increasing the risk of acceptor-side over-reduction at PSI and long-lived photoinhibition; too much ΔpH—or ΔpH that relaxes too slowly after a downshift—carries unnecessary NPQ into shade and suppresses ${\text{Φ}}_{PSII}$ and CO_2_ uptake (Zivcak et al., 2015; Allahverdiyeva et al., 2015; Tikhonov, 2018; Bethmann et al., 2019; Didaran et al., 2024). The same levers that shape pmf therefore set where the leaf sits on the photoprotection–productivity trade-off: anion flux pathways (e.g., VCCN1/CLCe) dissipate Δψ and thereby favor rapid ΔpH formation during light increases; KEA3 together with $g\left(H^{+}\right)$ sets the pace of ΔpH relaxation after transitions to lower light; and the balance between linear and cyclic electron flow determines how much pmf is generated in the first place. Because thylakoid architecture can impose spatial heterogeneity—microdomain ΔpH near b_6_f/PSII versus ATP synthase–rich regions—local kinetics can shape how quickly protection is engaged and how quickly antennae reopen (Armbruster et al., 2016; Wang and Shikanai, 2019). Viewed this way, performance is a timing problem rather than a maximization problem: protection benefits from a prompt ΔpH pulse during light upshifts, whereas productivity benefits from timely ΔpH relaxation during downshifts so that quenching does not outlast the need for it (Werdan et al., 1975; Wilson et al., 2021).

### 7.2 Operating-regime map

The operating-regime map that follows turns the proton-centric model into an experimenter’s field guide. For each common context in intact leaves, it links how the pmf is partitioned between ΔpH and Δψ, which controllers and effectors are expected to respond (KEA3, VCCN1/CLCe, ATP-synthase $g_{H^{+}}$, PsbS-mediated qE, and ΔpH-gated control at cytochrome *b*_6_f), and what carbon outcomes should result, including assimilation rate A, ${\text{Φ}}_{PSII}$, and the redox status and safety of PSI (Wang and Shikanai, 2019; Bru et al., 2021). The map is organized around regimes that recur in practice: seconds-scale sunflecks and shadeflecks; dark-to-light induction; periods of high sink strength such as elevated CO_2_; counter-ion–limited genotypes (vccn1, kea3, clce); situations with high ATP bias in photorespiring C_3_ leaves; TPU/Pi limitation mediated by triose-phosphate export and phosphate return; state-transition–driven antenna remodeling; low-temperature, high-light conditions that permit qH; methodological pitfalls in pmf partitioning such as Δψ misassignment; and proton geography in which microdomain ΔpH governs local kinetics.

For each light condition, **Table 3** specifies what balance of ΔpH and Δψ to expect, which protective and regulatory switches should engage, how net CO_2_ assimilation (A) is likely to behave, and which measurements resolve remaining ambiguities. The minimal, comparable measurement set includes ECSt as a pmf proxy; the ΔpH fraction from multi-wavelength ECS; $g_{H^{+}}$ from DIRK analysis; NPQ and P700 kinetics; A_Ci_ curves; xanthophyll de-epoxidation state; and Pi/TPT status where TPU is suspected (**Table 3**).

**Table 3:** pH-anchored operating regimes linking ΔpH/Δψ balance to ion-flux controllers and ATP-synthase $g_{H^{+}}$, and to expected NPQ/*b*_6_f behavior and assimilation performance; diagnostic readouts listed for each regime.

Light condition	Expected pmf partition	Effector/controller response	Carbon/physiology outcome	Primary diagnostics to run	Reference
Sunfleck onset (low to high light; seconds)	ΔpH rises rapidly; Δψ relieved if anion flux is effective	qE turns on within seconds via PsbS; ΔpH-gated control at b_6_f throttles PQH_2_ oxidation; PSI remains oxidized if Δψ relief is fast	PSI protected; A ramps as CBB enzymes and RCA activate	ECSt (pmf), ΔpH fraction, NPQ/P700 kinetics; verify VCCN1/CLCe function; ensure g_(H^+^)_ not excessive	Unified framework and partitioning logic; KEA3/VCCN1/CLCe roles and sunfleck behavior (Dukic et al., 2019)
Shadefleck onset (high to low light; tens of seconds)	ΔpH should relax promptly; Δψ minimal	qE relaxes on 30–60 s scale if KEA3 and ATP synthase are permissive	Φ_PSII_ recovers; A rebounds without unnecessary losses	g_(H^+^)_ (DIRK), ΔpH decay, NPQ half-off, KEA3 status; ZE activity	KEA3 as ΔpH relaxation valve; kinetics and design principles for qE/qZ (Armbruster et al., 2014).
Dark to light induction (minutes)	Initial Δψ transient converts to ΔpH as counter-ions move	Rapid qE onset; progressive activation of CBB (FBPase, SBPase, PRK) and RCA under alkaline stroma	Induction lag in A until stromal pH/Mg²^+^/redox reset; then steady increase	ECSt/ΔpH build kinetics, stromal pH proxies, A–time course; P700 redox	Proton-driven “switch-on” of CBB; RCA redox/ATP coupling; synthesis view (Harbinson and Hedley, 1993)
High sink strength (elevated CO_2_ or strong export)	ΔpH moderate; Δψ relieved quickly	Smaller qE amplitude; PSI stays oxidized	Faster A ramp; higher steady A at given light	A_Ci_, P700^+^ fractions, ΔpH fraction	From protons to carbon: sink effects on transients; unified “scorecard.” (Zhang et al., 2025)
Counter-ion–limited genotypes (vccn1/kea3/clce)	Δψ persists; ΔpH under-forms	Delayed qE; weaker ΔpH-dependent b_6_f ‘photosynthetic control’ during upshifts; PSI at risk during up-steps	PSI photoinhibition; suppressed A across days	Multi-λ ECS to confirm Δψ contamination; genotype checks; P700	KEA3–VCCN1–CLCe single to triple mutant logic; Δψ relief as prerequisite (Armbruster et al., 2014; Dukic et al., 2019).
High ATP-bias demand (photorespiring C_3_; C_4_ bundle sheath)	pmf boosted by CEF; ΔpH maintained	qE present but right-sized; NDH/PGR5 routes dominate as needed	A sustained at given light via better ATP/NADPH match	CEF markers (NDH, PGR5), ΔpH fraction, A	*In vivo* NDH-CEF dominance in C_4_ BS; “alternative power lines” synthesis (Cramer et al., 2011; Bellasio and Ermakova, 2022).
TPU/low-Pi limitation	pmf can be large but ATP synthase throttled by Pi scarcity	qE persists; stronger ΔpH-dependent b_6_f control ; ΔpH may linger	A plateaus early; slow post-fleck recovery	A_Ci_ with TPU signature; leaf Pi; TPT expression; ECSt vs A	Envelope transport (TPT), Pi feedback on ATP synthase and assimilation (Carstensen et al., 2018; McClain et al., 2023).
State transitions active (low–moderate light; changing spectra)	Partition varies locally as antenna repartitions	STN7-dependent redistribution; residual STN7-linked quenching modifies local demand	Subtle changes in Φ_PSII_ and PSI excitation balance	Fluorescence state-transition assays; structural readouts of grana size	Spatial remodeling and STN7-dependent quenching routes (Minagawa, 2011).
Low-temperature + high-light (qH-permissive)	Sustained ΔpH; Δψ modest	qH (PsbS-independent, LCNP/SOQ1-regulated) engages; slow to relax	Prolonged NPQ can cap Φ_PSII_ and A until ROQH1 resolves	NPQ components (qE vs qH), DEPS, recovery kinetics	qH mechanism and SOQ1/LCNP axis; timing relative to ΔpH (Bru et al., 2022).
Proton geography effects (domain heterogeneity; seconds)	Local ΔpH higher near b_6_f/PSII; sinks near ATP synthase	Faster protection at margins; faster reopening where ATP synthase concentrated	Domain-averaged signals hide microdomains; A depends on local timing	Pair ECS with spatial readouts (pH sensors, imaging)	Spatial–temporal organization and remodeling under light (Rieger et al., 2021; Ueno et al., 2025).

A, CO_2_ assimilation; ECSt, light-off ECS amplitude (pmf proxy); g_(H^+^)_, ATP-synthase proton conductance (DIRK); DEPS, xanthophyll de-epoxidation state; TPT, triose-phosphate/phosphate translocator; TPU, triose-phosphate utilization; BS, bundle sheath; CBB, Calvin–Benson–Bassham cycle; P700, PSI redox.

### 7.3 From lumen–stroma pH to pmf partitioning across light regimes

**Figure 4:**
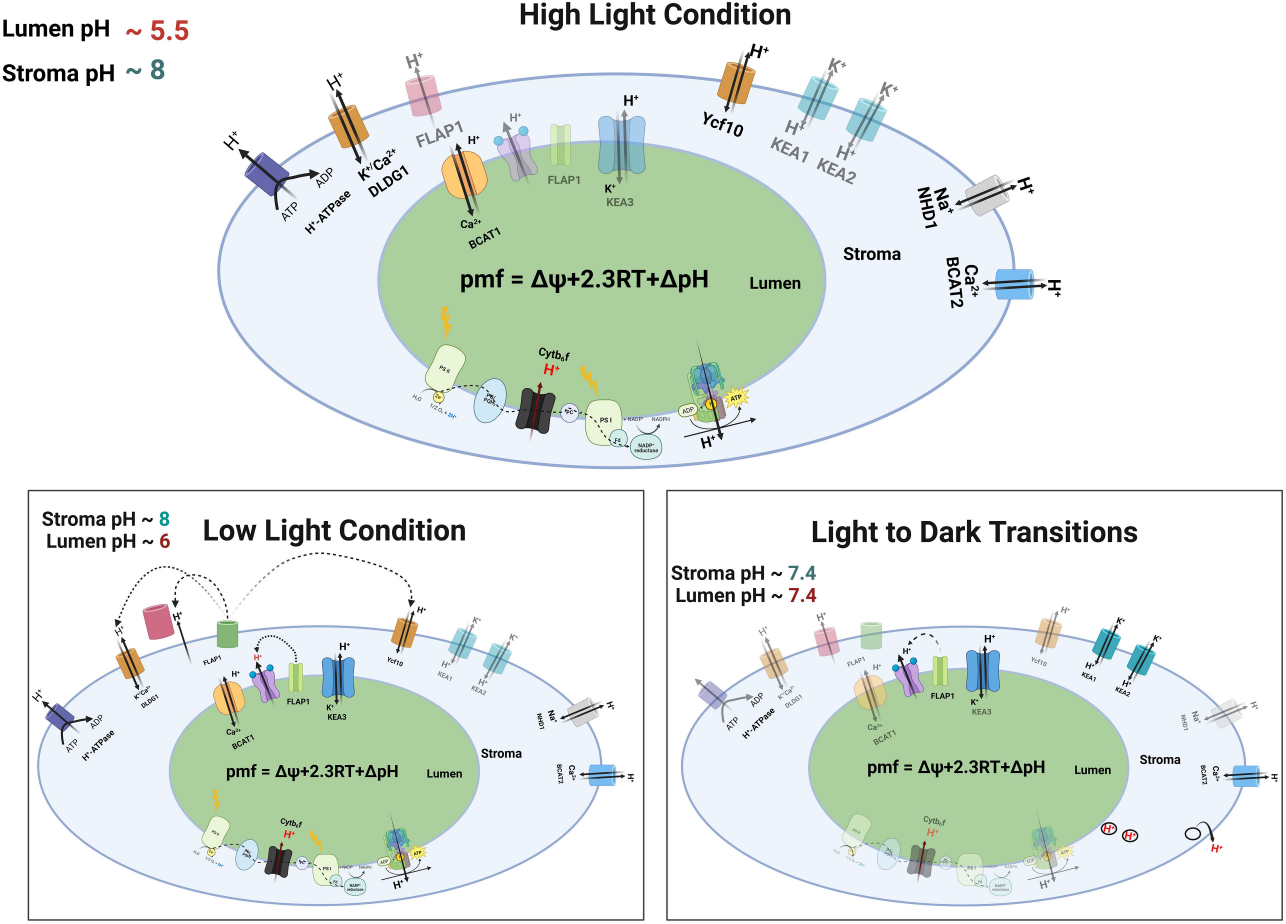
pH-anchored operating regimes that set pmf partitioning and ion-flux engagement. Three thylakoid states depicted with their characteristic pH set points and the corresponding control logic for the protonmotive force, pmf = Δψ + 2.303 RT/F · ΔpH. Top panel: High light conditions produce strong proton deposition into the lumen and an alkaline stroma (lumen pH ≈ 5.5; stroma pH ≈ 8.0). Anion influx through VCCN1/CLCe keeps the electrical term small, so pmf resides largely as ΔpH (Hagino et al., 2022). This favors rapid PsbS-mediated qE, VDE activity at its acidic optimum, and ΔpH-gated photosynthetic control at cytochrome b_6_*f, while ATP synthase provides regulated H^+^ efflux. Bottom left panel: Low light conditions stabilize the lumen closer to pH ≈ 6.0 with stroma near pH ≈ 8.0, yielding a smaller chemical term and a larger contribution from Δψ unless anion channels dissipate it. KEA3 activity increases to relax ΔpH after light decreases, limiting unnecessary NPQ carry-over and supporting recovery of PSII efficiency (Johnson and Ruban, 2011; Correa-Galvis et al., 2016; Kromdijk and Walter, 2023). Bottom right panel: Light to dark shows both compartments near pH ≈ 7.4, so the chemical term collapses and Δψ decays; NPQ dissipates as antennae reopen and ATP synthase no longer harvests pmf (Cruz et al., 2001; Hoh et al., 2024).

An operating-regime map is most informative when anchored to realistic pH set points that translate directly into the chemical component of the pmf. In intact leaves, illumination typically drives the stroma toward alkaline values near pH 8 while acidifying the lumen. Under strong light the lumen approaches roughly pH 5.5, whereas moderate light yields values nearer pH 6.0, and both compartments relax toward ~7.4 during light-to-dark transitions. Converting these pairs with (2.303 RT/F) ΔpH gives a chemical term of about 148 mV for high light (ΔpH=2.5), 118 mV for low–moderate light (ΔpH=2.0), and essentially zero in darkness (Werdan et al., 1975; Herdean et al., 2016; Hagino et al., 2022). These lumen and stromal pH dynamics and provide a quantitative backdrop for the pmf bars in **Figure 4**, which stack the computed ΔpH contribution above an electrical base representing Δψ. *In vivo*, Δψ tends to be smaller in bright light, somewhat larger at moderate light, and negligible after darkening, because ion movements dissipate the field as conditions change (Cruz et al., 2001; Wilson et al., 2021) (**Figure 4**).

Under high light conditions, rapid water oxidation at PSII and a full Q-cycle at cytochrome b_6_f deposit protons into the lumen so that pmf is carried predominantly as ΔpH around the pH 5.5 set point. Anion influx through the thylakoid bestrophin-like channel VCCN1 relieves Δψ and thereby biases the partition toward ΔpH; plants lacking VCCN1 accumulate a larger electrical term and are more vulnerable during rapid light increases, consistent with the logic of the operating-regime map (Sacksteder et al., 2000; Li et al., 2002; Cramer et al., 2011). Under these conditions, ΔpH protonates PsbS within seconds and activates VDE near its acidic optimum, while ΔpH-gated “photosynthetic control” at b_6_f restricts plastoquinol oxidation until carbon sinks are fully engaged, protecting PSI during the ramp (Degen and Johnson, 2024).

At low and moderate light, lumen pH stabilizes nearer pH 6.0, the chemical term is smaller, and Δψ contributes a larger fraction of the pmf unless it is actively dissipated. KEA3 becomes important for shaping this regime: by exchanging stromal K^+^ for luminal H^+^, KEA3 hastens ΔpH relaxation after down-steps, limits needless carry-over of qE, and improves PSII quantum efficiency in fluctuating light; genetic and overexpression analyses show that tuning KEA3 levels or regulation speeds recovery from high to low light without sacrificing the capacity to protect during the next up-step (Armbruster et al., 2016; Wang and Shikanai, 2019). Because stromal pH remains near pH 8 in this regime, Calvin-cycle enzymes stay in a light-activated state, but the weaker acidification means that effective Δψ relief is essential to prevent PSI over-reduction when light suddenly increases (Michelet et al., 2013).

During light-to-dark transitions, both compartments relax toward pH 7.4, the ΔpH term collapses, and Δψ decays, extinguishing the driving force for ATP synthesis and allowing NPQ to dissipate. Inner-envelope K^+^/H^+^ antiporters KEA1 and KEA2 are now fully engaged to neutralize the stroma, complementing thylakoid-level H^+^ efflux and explaining the rapid loss of the electrochromic signal at darkening; functional analyses show that KEA1/KEA2 activity is dispensable for maintaining alkaline stroma in the light but is critical for fast down-regulation of stromal pH in the dark, in line with the behavior depicted in the schematic (Correa Galvis et al., 2020; Dukic et al., 2022; Juhaszova et al., 2022).

Partitioning matters because the two components of pmf have distinct physiological consequences. A larger ΔpH fraction promptly engages qE and *b*_6_f control and is therefore inherently protective during up-steps, whereas an oversized or persistent Δψ-dominant pmf is problematic because it fails to generate the ΔpH signal needed for protective feedbacks (Tikhonov, 2018; Wilson et al., 2021). Experimental manipulations that elevate the electrical term at the expense of ΔpH increase PSII damage and depress productivity under fluctuating light, underscoring why leaves are wired to counter-ion flux limits Δψ build-up, thereby allowing proton pumping to manifest primarily as ΔpH during illumination and to release ΔpH quickly when light falls (Cruz et al., 2001).

Electrochromic-shift spectroscopy remains the standard for partitioning pmf and estimating ATP-synthase proton conductance *in vivo*, but quantitative use requires spectral controls and reporting conventions so that Δψ estimates are not confounded by overlapping pigment signals; coupling ECS with fluorescence-based NPQ kinetics and P700 redox traces allows the ΔpH-dependent switches and PSI safety to be operated alongside the partition itself (Johnson, 2011; Brestic et al., 2015; Tikkanen et al., 2015; Kono et al., 2017). Finally, structural work on the chloroplast ATP synthase provides a mechanistic basis for how proton efflux is gated during transients and explains why adjustments in $g_{H^{+}}$ markedly reshape ΔpH formation and decay across the three regimes (Johnson et al., 2014; Davis et al., 2017; Wilson et al., 2021).

## 8 Mechanistic ODE models of pmf partitioning as a quantitative complement to the proton-circuit framework

The proton-circuit framework used throughout this review is intentionally modular: proton deposition by LEF/CEF builds pmf, ATP synthase spends pmf as ATP with an effective proton conductivity g(H^+^), and counter-ion pathways reshape how much of the instantaneous pmf is expressed as ΔpH versus Δψ. While this conceptual circuit is useful for interpreting light-transient phenotypes, many of its key internal states (Δψ, lumen pH, and individual ion fluxes) are only partially observable *in vivo* and often must be inferred from composite signals such as the electrochromic shift (ECS), chlorophyll fluorescence, and P700 redox kinetics. Mechanistic ordinary differential equation (ODE) models provide a compact way to formalize these couplings, test whether a given mechanistic interpretation is self-consistent, and quantify how changes in specific conductances (e.g., ATP synthase or ion pathways) are expected to reshape the timing of ΔpH formation and relaxation under defined light regimes. Recent ODE-based studies—particularly the series by Lyu et al. explicitly simulate Δψ, ΔpH, and pmf during light transitions while incorporating key physical features such as thylakoid ion currents and membrane-surface ion binding, offering an important systems-level complement to experimental approaches (Lyu and Lazár, 2017, 2022; Lyu and Lazár, 2023; Lyu and Lazár, 2024a, b).

### 8.1 Mapping the proton circuit to state variables and flux terms

Most ODE formulations relevant to this review share a common “state-flux” structure. The internal state can be represented by a small set of time-dependent variables, including lumen proton activity (often expressed as lumen pH), trans-thylakoid electric potential (Δψ), and total pmf (Δp = Δψ + (2.303RT/F)ΔpH), together with optional redox states (e.g., PQ pool, PSI acceptor side) depending on model scope. These states evolve as the net result of fluxes that correspond directly to the circuit elements discussed in Sections 2–7:

- Proton deposition (source term): a light-dependent proton influx to the lumen driven by LEF and augmented by CEF (with pathway-specific contributions depending on whether PGR5/PGRL1 and/or NDH are represented).
- Proton efflux (valve term): proton return through ATP synthase, typically parameterized as a conductance or kinetic term that is closely related to experimentally estimated g(H^+^).
- Charge-compensation and partitioning (shaper terms): ionic currents (anion influx and/or cation efflux) and exchange processes (e.g., K^+^/H^+^ antiport) that dissipate Δψ and thereby control how efficiently continued proton pumping is converted into ΔpH.

In a minimal dynamical representation, lumen acidification can be expressed conceptually as:

(Equation 2):


$$\frac{d\left(\text{Δ}pH\right)}{dt}\propto \frac{J_{H^{+}}^{pump}-J_{H^{+}}^{ATPase}-J_{H^{+}}^{leak/antiport}}{\beta_{lumen}}$$

and Δψ can be treated as an electrical state set by a membrane capacitance and net ionic currents:

(Equation 3):


$$\frac{d\left(\text{Δ}\psi \right)}{dt}\;\propto \;\frac{I_{net}}{C_{m}}$$

where $J_{H^{+}}^{pump}$ aggregates LEF/CEF-linked proton deposition, $J_{H^{+}}^{ATPase}$ reflects ATP synthase proton efflux (linked to g(H^+^) and substrate availability), $I_{net}$ captures the balance of proton-generated charge separation and counter-ion currents (e.g., Cl^-^ influx through VCCN1/CLCe-like terms and cation fluxes), $\beta_{lumen}$ is an effective lumen buffering capacity, and $C_{m}$ is an effective thylakoid membrane capacitance. Although different models implement these terms at different levels of detail, the central advantage is that the framework forces explicit accounting of how fluxes and constraints jointly determine ΔpH:Δψ partitioning over time (Lyu and Lazár, 2017, 2022).

### 8.2 What ODE models add beyond qualitative circuit logic

A primary contribution of mechanistic ODE models is that they separate three concepts that are easily conflated in narrative descriptions: (i) total pmf magnitude, (ii) pmf parsing into ΔpH versus Δψ, and (iii) the kinetics with which parsing changes during transients. By explicitly representing the competing timescales of proton pumping, ATP synthase discharge, and counter-ion flux, models can reproduce situations where pmf rises quickly but lumen acidification is delayed (Δψ-heavy transient) or where ΔpH persists because ATP synthase throughput is limited by kinetic regulation and/or substrate supply (Lyu and Lazár, 2017, 2023).

A second contribution is that several recent ODE formulations incorporate electrostatic features that are difficult to isolate experimentally, including membrane-surface ion binding and Donnan/diffusion components of electric potential. These features provide a mechanistic route for Δψ behavior that is not simply a “short-lived spike,” and they offer quantitative hypotheses for when electrical contributions could persist or become functionally consequential despite substantial ΔpH expression (Lyu and Lazár, 2022).

Finally, recent work has emphasized sensitivity analysis as a practical bridge between modeling and experiment: by ranking which parameters most strongly influence predicted Δψ/ΔpH dynamics or observable signatures (e.g., ECS or fluorescence kinetics), models can guide which measurements are most informative and which engineering interventions are most likely to shift leaf performance in targeted regimes (Lyu and Lazár, 2024a, b).

### 8.3 Representative ODE modeling efforts relevant to pmf partitioning

To keep this section focused, **Table 4** highlights representative ODE-based studies that explicitly simulate Δψ, ΔpH, and pmf dynamics while exploring the influences of ion fluxes, ATP synthase activity, and membrane electrostatics. These studies are not intended as an exhaustive modeling review; rather, they exemplify how formal dynamical models can be used to (i) test the internal consistency of mechanistic interpretations of ECS/fluorescence/P700 data, (ii) quantify how changing conductances shifts ΔpH “timing,” and (iii) identify parameter regimes where ΔpH-gated control might decouple from expected photoprotective outcomes.

**Table 4:** Representative mechanistic ODE models relevant to thylakoid pmf partitioning and its regulation.

Primary focus	Key circuit levers represented	Outputs most relevant here	Reference
ODE simulation of light-driven pmf dynamics	Proton pumping, ATP synthase discharge, counter-ion effects (effective currents/terms)	Δψ, ΔpH, pmf time courses under light changes	(Lyu and Lazár, 2017)
Electrostatic components and ion binding in pmf parsing	Adds membrane-surface ion binding/Donnan-like contributions plus ion flux terms	Δψ components, ΔpH/pmf dynamics, links to ECS/P515 interpretations	(Lyu and Lazár, 2022)
Ion-flux control of Δψ/ΔpH dynamics	Emphasizes ion flux pathways and coupling to pmf partition	Predicted sensitivity of Δψ/ΔpH to ion conductances	(Lyu and Lazár, 2023)
ATP synthase modulation and pmf partition	Emphasizes ATP synthase activity as a dynamical control node	pmf, Δψ/ΔpH trajectories under altered ATP synthase behavior	(Lyu and Lazár, 2024a)
Joint simulation of optical signatures	Integrates pmf dynamics with optical readouts (fluorescence/ECS-like)	Observable kinetics linked to internal states	(Lyu and Lazár, 2024a)
Parameter sensitivity/screening	Systematic sensitivity (e.g., Morris-type)	Ranked parameter influence on outputs	(Lyu et al., 2024b)

## 9 Conclusion

This review describes a proton-centric view of leaf photosynthesis in which the partitioning of the thylakoid proton motive force (pmf)—the balance between ΔpH and Δψ—links light harvesting, ATP synthesis, photoprotection, and downstream carbon metabolism. Proton pumps in LEF and CEF build pmf; ATP synthase and thylakoid ion pathways (KEA3, VCCN1, CLCe) shape how much pmf is expressed as lumen acidification and how rapidly that acidification forms and relaxes; and pH-sensitive effectors translate lumen acidification into rapid qE and ΔpH-gated control at cytochrome b_6_f, safeguarding PSI during transients. Stromal alkalinization, Mg²^+^ mobilization, and thioredoxin activation contribute to “switching on” Calvin–Benson–Bassham (CBB) enzymes, while CEF and metabolite shuttles help balance ATP/NADPH. Crucially, carbon metabolism is not merely a downstream “consumer”: stromal metabolite pools—especially ADP and inorganic phosphate (Pi), and their recycling via export/TPU constraints—feed back on ATP synthase throughput and therefore on how quickly pmf is spent and how long ΔpH-dependent regulation persists.

In fluctuating light, photosynthetic performance can often be understood less as a problem of maximizing pmf or NPQ capacity and more as a problem of timing: leaves need to generate sufficient ΔpH quickly during light up-steps to protect PSI, and then relax ΔpH in a timely manner during down-steps so that quenching does not unnecessarily suppress CO_2_ assimilation. Within this framework, the operating-regime map—together with a common diagnostic set (multi-wavelength ECS for ΔpH/Δψ and g(H^+^), NPQ and P700 kinetics, and gas exchange)—provides concrete, testable hypotheses and a practical basis for making future datasets more directly comparable across studies and species.

A key implication of this synthesis is that a proton−circuit framework is most powerful when treated as a quantitative organizing principle with explicit boundary conditions, rather than a universal, single−variable explanation of leaf performance. pmf and its ΔpH:Δψ partitioning provide a mechanistic bridge between light reactions, rapid photoprotective switching, and ATP production, but the realized ΔpH kinetics and downstream outcomes emerge from coupled physical constraints and metabolic feedbacks in intact leaves. Stromal metabolite pools—especially ADP and inorganic phosphate (Pi)—can directly modulate ATP synthase throughput (g(H^+^)) and thus the rate at which pmf is converted into ATP or retained as lumen acidification, feeding back on both ΔpH−dependent regulation and CO_2_ assimilation. Accordingly, strengthening the integration of proton−centric diagnostics with Pi/adenylate status and sink activity (and, where feasible, incorporating these variables into quantitative models) will be essential for identifying when ΔpH timing is the dominant determinant of protection–productivity trade−offs and when additional metabolic or regulatory layers set the limiting behavior (**Table 4**).
